# Crosstalk between TBK1/IKKε and the type I interferon pathway contributes to tubulointerstitial inflammation and kidney tubular injury

**DOI:** 10.3389/fphar.2022.987979

**Published:** 2022-09-23

**Authors:** Gina Córdoba-David, Jorge García-Giménez, Regiane Cardoso Castelo-Branco, Susana Carrasco, Pablo Cannata, Alberto Ortiz, Adrián M. Ramos

**Affiliations:** ^1^ Department of Nephrology and Hypertension, IIS-Fundación Jiménez Díaz, Universidad Autónoma de Madrid, Madrid, Spain; ^2^ RICORS 2040, Madrid, Spain; ^3^ Department of Pathology, IIS-Fundación Jiménez Díaz, Universidad Autónoma de Madrid, Madrid, Spain; ^4^ Department of Medicine, Universidad Autónoma de Madrid, Madrid, Spain

**Keywords:** TBK1/IKKε, type I interferon, kidney injury, TWEAK, LPS, inflammation, cell death

## Abstract

The type I interferon (TI-IFN) pathway regulates innate immunity, inflammation, and apoptosis during infection. However, the contribution of the TI-IFN pathway or upstream signaling pathways to tubular injury in kidney disease is poorly understood. Upon observing evidence of activation of upstream regulators of the TI-IFN pathway in a transcriptomics analysis of murine kidney tubulointerstitial injury, we have now addressed the impact of the TI-IFN and upstream signaling pathways on kidney tubulointerstitial injury. In cultured tubular cells and kidney tissue, IFNα/β binding to IFNAR activated the TI-IFN pathway and recruited antiviral interferon-stimulated genes (ISG) and NF-κB-associated proinflammatory responses. TWEAK and lipopolysaccharide (LPS) signaled through TBK1/IKKε and IRF3 to activate both ISGs and NF-κB. In addition, TWEAK recruited TLR4 to stimulate TBK1/IKKε-dependent ISG and inflammatory responses. Dual pharmacological inhibition of TBK1/IKKε with amlexanox decreased TWEAK- or LPS-induced ISG and cytokine responses, as well as cell death induced by a complex inflammatory milieu that included TWEAK. TBK1 or IRF3 siRNA prevented the TWEAK-induced ISG and inflammatory gene expression while IKKε siRNA did not. *In vivo*, kidney IFNAR and IFNβ were increased in murine LPS and folic acid nephrotoxicity while IFNAR was increased in human kidney biopsies with tubulointerstitial damage. Inhibition of TBK1/IKKε with amlexanox or IFNAR neutralization decreased TI-IFN pathway activation and protected from kidney injury induced by folic acid or LPS. In conclusion, TI-IFNs, TWEAK, and LPS engage interrelated proinflammatory and antiviral responses in tubular cells. Moreover, inhibition of TBK1/IKKε with amlexanox, and IFNAR targeting, may protect from tubulointerstitial kidney injury.

## Introduction

Aberrant activation of innate immunity may lead to maladaptive inflammation and tissue damage during infection and even under sterile pathological conditions. Type I-interferons (TI-IFNs), i.e., interferon-alpha (IFNα) and interferon-beta (IFNβ), are immunomodulatory cytokines typically involved in antiviral responses, which also modulate bacterial or fungal infections (Bogdan et al., 2004; McNab et al., 2015). TI-IFN responses are recruited by structurally conserved pathogen-associated molecular patterns (PAMPs) or host-derived damage-associated molecular patterns (DAMPs). Both PAMPs and DAMPs activate cytoplasmic (RIG, AIM2, cGAS), or membrane (TLR family) receptors which phosphorylate the TBK1/IKKε tandem of noncanonical IκB kinases (IKKs) leading to phosphorylation of interferon regulatory factor (IRFs) transcription factors, such as IRF3 and IRF7, to promote the synthesis of TI-IFNs (Fitzgerald et al., 2003; Hertzog et al., 2003; Honda & Taniguchi, 2006; Crowl et al., 2017).

Binding of TI-IFNs to interferon-α/β receptor (IFNAR) induces autophosphorylation of associated JAK proteins (e.g., TYK2, JAK1), leading to STAT1 and STAT2 phosphorylation/activation to induce the transcription of an extensive set of interferon-stimulated genes (ISGs) (Platanias, 2005; Lee & Ashkar, 2018). Overall, the ISG response regulates diverse key cellular processes including apoptosis, autophagy, proliferation, differentiation, and affects the early and late stages of viruses’ life cycle. However, specific roles have only been identified for a few ISGs (Schneider et al., 2014). Crosstalk between the TI-INF pathway and NF-κB (which play a critical role in kidney inflammation) modulates pro-inflammatory transcription and promotes cell survival in human and murine IFNAR-bearing cells, and controls viral infection *in vivo* (Yang et al., 2000; Sanz et al., 2010b; Rubio et al., 2013; Piaszyk-Borychowska et al., 2019). TBK1 and IKKε also control NF-κB inducers (Shin & Choi, 2019). IRF homodimers or heterodimers cooperate with NF-κB to promote the synthesis of some NF-κB-dependent chemokines, typically Cxcl10, while NF-κB cooperates with IRF3/7 to promote IFNβ synthesis and direct (not dependent on STAT signaling) ISG transcription (Honda & Taniguchi, 2006; Freaney et al., 2013; Schneider et al., 2014; Iwanaszko & Kimmel, 2015). However, whether these interactions contribute to regulating kidney inflammation is unclear, despite the observation that human diseases associated with kidney injury, e.g. viral infections, type I interferonopathies, and autoimmune conditions (e.g., systemic lupus erythematosus) are characterized by enhanced IFN-I signaling (Lodi et al., 2022). Thus, although some reports have highlighted the contribution of T1-IFNs in postischemic kidney injury or lupus nephritis (Freitas et al., 2011; Ding et al., 2021), the regulation and function of molecular routes upstream or downstream of TI-IFN remain mostly unexplored in intrinsic kidney cells and kidney injury, especially in tubular cells and tubulointerstitial disease.

TNF-like weak inducer of apoptosis (TWEAK/TNSF12) is a TNF superfamily cytokine that promotes tubular cytokine synthesis, tubular proliferation, and tubulointerstitial inflammation. TWEAK binding to the fibroblast growth factor-inducible 14 (FN14; TNF receptor superfamily member 12a [TNFRSF12a]) receptor activates classical and alternative pathways of NF-κB involving canonical IKKs (IKKα, IKKβ, and IKKγ (NEMO)) [(Poveda et al., 2013)]. Moreover, in the presence of TNFα and IFNγ, TWEAK triggers apoptosis in tubular cells (Justo et al., 2006; Sanz et al., 2011). Therefore, TWEAK/Fn14 axis blockade decreases kidney inflammation and injury in acute kidney injury (AKI) and autosomal dominant polycystic kidney disease (ADPKD) (Sanz et al., 2008; Martin-Sanchez et al., 2018; Cordido et al., 2021).

We now describe new molecular pathways activated by TWEAK and LPS involving non-classical IKKs (TBK1 and IKKε) and IRF3, all of which regulate proinflammatory NF-κB activity and trigger the TI-IFN pathway in tubular cells and contribute to kidney inflammation and injury.

## Material and methods

### Ethic statements

Procedures on animals were performed according to the European Community and Animal Research Ethical Committee guidelines and were approved by the IIS-FJD Animal Research Ethical Committee and the Consejería de Medio Ambiente y Ordenación del Territorio, Comunidad de Madrid (PROEX 038/19).

The study with human samples complied with ethical precepts formulated in Order SAS 3470/2009 and the Declaration of Helsinki of the World Medical Association on ethical principles for medical research and were approved by the institutional Research Ethical Committee (PIC026-19-FJD). Samples of patients were requested through written informed consent and collected under a biobank regimen.

### Cell culture

Murine MCT cells are a well-characterized cell model of kidney tubular epithelium suitable to study molecular mechanisms of kidney injury (Haverty et al., 1988). MCT cells were grown in RPMI 1640 (GIBCO, Grand Island, NY) supplemented with 10% decomplemented fetal bovine serum (DFBS), 2 mM glutamine, 100 U/mL penicillin, and 10 mg/ml streptomycin, in 5% CO_2_ at 37°C. For experiments, cells were stimulated with 100 ng/ml human TWEAK; 0.01 to 100 mUI/ml IFNα and IFNβ (R&D Systems Inc.); the cytokine mixture made of 100 ng/ml human TWEAK, 30 ng/ml TNFα, and 30 U/ml interferon-γ (IFNγ, PeproTech) or LPS (100 ng/ml, Merck). The following chemical inhibitors were used: 10 μM PF-06700,841 tosylate salt (Sigma-Aldrich, Merck); 50 μM amlexanox; 10 μM Parthenolide, and 2.5 μM IKK16 (MedChemExpress). All the inhibitors were added to cultured cells 1 h before the stimuli. Stock solutions of the stimuli and inhibitors were made according to the specified manufacturers’ instructions. Cells were also treated with the IFNAR neutralizing antibody for 3 h before the stimuli.

### Gene and protein expression assays

Gene transcription was analyzed through quantitative reverse transcription PCR (qRT-qPCR) by using predesigned gene expression assays (TaqMan^®^, Applied Biosystems-Termofisher Scientific, Waltham, MA, United States). Proteins were assessed by western blot, ELISA, and immunocytochemical-immunofluorescence assays. Standard procedures were applied.

### Western blot

Samples were homogenized in lysis buffer (50 mmol/L Tris, 150 mmol/L NaCl, 2 mmol/L EDTA, 2 mmol/L EGTA, 0.2% Triton X-100, 0.3% NP-40, 0.1 mmol/L PMSF, 25 mmol/L NaF). Proteins were separated by 10% SDS-PAGE under reducing conditions, then blotted onto nitrocellulose membranes. Membrane blockade was accomplished with 5% defatted milk in TBS-T (0.05 mol/L Tris, 0.15 mol/L NaCl, 0.05% Tween 20, pH 7.8). Thereafter, membranes were overnight probed at 4°C with primary antibodies in the same blocking solution or 5% BSA in TBS-T and then incubated with secondary HRP-conjugated antibodies for 1 h at room temperature. The following primary antibodies were used to detect specific proteins of interest: rabbit polyclonal anti-p-STAT1 (Tyr701) (Invitrogen, 44-376G), p-TYK2 (pTyr1054) (Origene, TA333304), and p-IKKε (Ser172) (Sigma Aldrich, 06-1340); rabbit monoclonal anti-p-TBK1/NAK (S172) (D52C2) XP^®^ (Cell Signaling Technology, 1,675,483), TBK1/NAK (E8I3G) (Cell Signaling Technology, 38,066), IKKε (D61F9) XP^®^ (Cell Signalling Technology, 3416), pIRF3 (Ser396) (4D4G) (Cell Signalling Technology, 4,947) and IRF-3 (D83B9) (Cell Signalling Technology, 4,302); monoclonal mouse anti-pIKBα (Santa Cruz, sc-8404). Anti-α-Tubulin (Sigma-Aldrich, MAB374) and anti-GAPDH (Millipore, MAB374) were used to assess protein loading homogeneity.

### Immunofluorescence

Cells plated onto glass coverslips were fixed in 4% paraformaldehyde and permeabilized in 0.2% Triton X-100/PBS, washed in PBS, and overnight incubated with polyclonal rabbit anti-p-IRF3 (pSer396) antibody (1:50, Sigma-Aldrich, SAB4504031 or anti-p65 (1:100; Santa Cruz Biotechnology, sc-8008) followed by Alexa 488-conjugated secondary antibody (1:300; Invitrogen) (3). Nuclei were counterstained with DAPI.

### ELISA

Ccl5 expression levels in the supernatants of cultured cells subjected to proinflammatory stimulation were assessed by ELISA (DuoSet ELISA Kit, R&D Systems, Minneapolis, MN) according to the manufacturers’ instructions.

### siRNA transfection

MCT cells were grown in six-well plates and transfected with a mixture of a set of three specific siRNA for TBK1 (75 nM), IKKε (75 nM), or IRF3 (40 nM) (Stealth RNAi™, Invitrogen-TermoFisher Scientific, MA) and Lipofectamine RNAiMAX Transfection Reagent (Invitrogen) made in Opti-MEM I Reduced Serum Medium. After 18 h, cells were washed and cultured for another 48 h in a complete medium containing 10% BSA, and finally serum-deprived for 24 h before stimulation with TWEAK, TTI or z/TTI to evaluate gene mRNA expression and cell death. A siRNA negative control (Stealth RNAi™, Invitrogen) with the same GC content of specific siRNA oligonucleotides was used as a control. After transfection, cells were stimulated at time points when protein expression was reduced by approximately 90%.

### *In vitro* and *in vivo* cell death assessment

For assessment of the overall death rate, cells were washed with PBS following stimulation and then incubated with 0.5 mg/ml MTT (Sigma, Merck) for 1 h at 37°C to detect changes in the metabolic activity. After this step, the MTT solution was withdrawn, and cells were allowed to air dry. Finally, deposits of reduced MTT were dissolved with DMSO, and their absorbance was read at 570 nm. *In vivo* cell death was assessed by a TUNEL assay performed in 3 µm-thick sections of paraffin-embedded renal tissue (*In Situ* Cell Death Detection Kit, Fluorescein, Roche Applied Science), according to the manufacturer’s protocol. TUNEL positive cells were counted in 10 randomly chosen fields with a fluorescent microscope.

### Animal models

Models were developed in wild-type0-12-week-old C57BL/6 mice (Charles River Chatillon-Sur-Charlaronne, France) or in *TLR4* ^*−/−*^ mice from the same genetic background (Dr S Akira’s laboratory, Osaka University, Japan and generously provided by Dr C. Guerri, CIPF, Valencia, Spain). Four different models of kidney inflammation and injury were assessed. 1) Systemic administration of IFNβ was conducted as previously published (Van-Holten et al., 2004) and dose (0.5 μg/mouse, IP) was established in dose-response test models evaluating renal inflammation. At the times chosen for the experiments (4 and 24 h), recombinant mouse IFNβ (R&D Systems, McKinley Place, MN) caused no tubular cell death or loss of renal function. 2) The TWEAK murine model was previously standardized in our laboratory. It displays renal inflammation originating from classical and alternative activation of NF-κB (Sanz et al., 2010a). Wild-type or *TLR4* ^*−/−*^ mice were challenged with recombinant human TWEAK (Merck, Darmstadt, Germany) and renal tissue was analyzed after 24 h. Additionally, some mice also received a second intervention: parthenolide (MedChemExpress, Monmouth Junction, NJ) (70 μg/mouse, IP) or amlexanox (Biorbyt, Cambridge, UK) (50 mg/kg) to inhibit NF-κB or TBK1/IKKε kinases respectively before TWEAK dosage. 3) Endotoxemia-induced kidney inflammation was assessed 24 h after the administration of 5 mg/kg IP bacterial lipopolysaccharide (LPS, Sigma) (LPS nephropathy, LPSN). The dose was chosen on the basis that it may induce a feasible inflammatory reaction and injury in the kidney without hemodynamic compromise and mortality (Panzer et al., 2009; Hato et al., 2019). TBK1/IKKε was inhibited with 50 mg/kg (PO) amlexanox whereas inhibition of the TI-IFN pathway was accomplished with 0.5 mg/kg/day (IP) neutralizing anti-IFNAR antibody (MAR1-5A3, Leinco Technologies). Inhibitors were administered 2 h before LPS. Rat IgG1 (MAB005, R&D Systems) was used as isotype control. 4) Murine folic acid-induced nephrotoxicity (FAN) is characterized by tubular cell death, leukocyte infiltration, and subsequent tubular regeneration that has been reported in humans (Metz-Kurschel et al., 1990; Martin-Sanchez et al., 2018; Yan, 2021). FAN was induced by a single IP injection of 250 mg/kg folic acid and analyzed at 24-96 h (Martin-Sanchez et al., 2018; Yan, 2021). Inhibition with amlexanox or anti-IFNAR antibody was scheduled 2 h before and 24 after FA injection.

Mice were euthanized under anaesthesia with 35 mg/kg ketamine (Ketolar/Pfizer) and 5 mg/kg xylazine (Rompun/Bayer). Blood for serum analytical assessment was drawn from the saphena vein and collected on tubes coated with EDTA. Plasma was obtained by centrifugation (1500 rpm, 5 min). Kidneys were perfused *in situ* with cold saline before removal. One kidney was snap-frozen in liquid nitrogen for RNA and protein studies and the other fixed and paraffin-embedded for immunohistochemistry.

Urea plasma levels were assessed by biochemical methods intended for automatic measurements based on the enzymatic decomposition with urease, then followed by colorimetric detection of the reaction product (Roche/Hitachi cobas^®^ c701/702).

### Transcriptomics arrays conditions and analysis

Transcriptomics arrays of kidney tissues from mice 24 h after folic acid or vehicle (*n* = 3/group) were previously published (González-Guerrero et al., 2018; Fontecha-Barriuso et al., 2019). Transcriptomics were performed at Unidad Genómica Moncloa, Fundación Parque Científico de Madrid, Madrid, Spain. Affymetrix microarray analysis followed the manufacturer’s protocol. Image files were initially obtained through Affymetrix GeneChip^®^ Command Console^®^ Software (AGCC) (Affymetrix, Thermo Fisher Scientific, Santa Clara, CA). Subsequently, robust multichip analysis (RMA) was performed using Affymetrix Expression Console^®^ Software Affymetrix, Thermo Fisher Scientific. Starting from the normalized RNA, a significance analysis of microarrays was performed using the limma package (Babelomics, www.babelomics.org), using a false discovery rate (FDR) of 5% to identify genes that were significantly differentially regulated between the analyzed groups. Canonical pathway enrichment analyses were performed using the public database Reactome (www.reactome.org) (supported by United States National Institutes of Health; Toronto University; European Union and the European Molecular Biology Laboratory) 34,788,843 (Gillespie et al., 2022) and Interferome (www.interferome.org) (Monash Institute of Medical Research, University of Cambridge) (Samarajiwa et al., 2009).

### Immunohistochemistry

Paraffin-embedded sections were stained using standard histology procedures. Immunostaining was performed in 3 μm thick tissue sections that were deparaffinized and antigen retrieved using the PT Link system (Dako Diagnostics, Barcelona, Spain) with Sodium Citrate Buffer (10 mM) adjusted to pH 6-9, depending on the marker. For colorimetric immunohistochemistry, endogenous peroxidase was blocked and then sections were incubated overnight at 4°C with the following primary antibodies: polyclonal rabbit anti-human CD3 (ready to use; DAKO A0452), anti-human MPO (ready to use; DAKO IS511); monoclonal mouse anti-mouse T-bet/Tbx21 [4B10] (1:200; Abcam ab91109); monoclonal rat anti-mouse F4/80 (1:50, MCA497, Bio-Rad); polyclonal rabbit anti-human IFNAR2 (1:100, LS-B13369, LSBio); polyclonal rabbit anti-human IFN beta (1:400, PA5-20390, TermoFisher). Finally, sections were washed, stained with 3,3′-diaminobenzidine (DAB) as chromogen (Dako, Denmark), counterstained with Carazzi`s hematoxylin, dehydrated, and mounted in DPX medium (Merck). Fluorescent immunohistochemistry was developed in tissue sections that were first permeabilized with 0.2% Triton X-100 for 5 min, then blocked with 4% BSA/10% species-specific serum (the same animal species that animal source of primary antibodies), followed by sequential primary and secondary antibodies incubation for 90 m or 60 m, respectively, and finally, the nuclei stained with DAPI for 5 m. Primary antibodies used were: polyclonal rat anti-mouse F4/80 (1:50, MCA497, Bio-Rad); FLEX monoclonal mouse anti-human CD31 (ready to use, GA610, clone JC70A DAKO); polyclonal rabbit anti-human IFN beta (1:100, PA5-20390, TermoFisher). Primary antibody binding to specific antigens was revealed by using the following secondary antibodies (1:200, Invitrogen): Alexa fluor donkey anti-rat 488 (A21208), goat anti-mouse 633 (A21050), goat anti-rabbit 633 (A21070), and donkey anti-rabbit 488 (A21206).

Images were obtained by optical (BX53F2 model, Olympus Spain) or confocal (SP5, Leica Microsystems, Spain) microscopy and quantified with ImageProPlus software (Media Cybernetics, Bethesda, MD). Results are shown as the number of positive cells from 10 randomly chosen fields per kidney (20–4×0 objective) or as the percentage of stained area, considering the total area as 100%. Negative controls include non-specific immunoglobulin and no primary antibody.

### Human samples

Biopsies from AKI patients (*n* = 6) and healthy kidney tissue from nephrectomy specimens (*n* = 4) were obtained from the IIS-Fundación Jimenez Diaz Biobank (IIS-Fundación Jimenez Diaz, Madrid, Spain). Clinical characteristics of human samples intended for this study are provided in Supplementary Table S1.

### Statistics

Statistical analysis was performed using GraphPad Prism (Dotmatics, San Diego, CA), expressing results as sem ± sd. Significance (*p* < 0.05) was assessed by a non-parametric Mann-Whitney test for two independent samples.

## Results

### Activation of upstream regulators of the TI-IFN pathway in mouse and human tubulointerstitial injury

Recent emphasis on the association of enhanced IFN-I signaling and kidney injury has focused on glomerular injury (Lodi et al., 2022), without considering associated tubulointerstitial injury. Pathway enrichment analysis in a previously reported transcriptomics dataset of murine folic acid nephropathy (FAN) (González-Guerrero et al., 2018; Fontecha-Barriuso et al., 2019) identified multiple activated upstream regulators of the TI-IFN pathway having an absolute z-score > 2.0 and *p*-value < 0.05 (Supplementary Table S2). Moreover, 168 of the 865 (19.4%) most upregulated genes (fold change ≥1.5 times; *p* and FDR <0.05) and 37 of the 333 (11.1%) most downregulated genes (fold change ≥0.5 times; *p* and FDR <0.05) were classified as interferon regulated genes (IRG) in the Interferome database, based on several datasets of mouse cells challenged with TI-IFNs. Subsequent bibliography-based expression profiling analysis also showed overexpression (*p* and FDR <0.05) of an ISG mRNA signature (Supplementary Table S3). Hence, kidney transcriptomics identified a direct activity of the TI-IFN pathway on ISG transcription or the propagation of an intricate signaling network resulting from the engagement of this pathway in murine tubulointerstitial injury.

**FIGURE 1:**
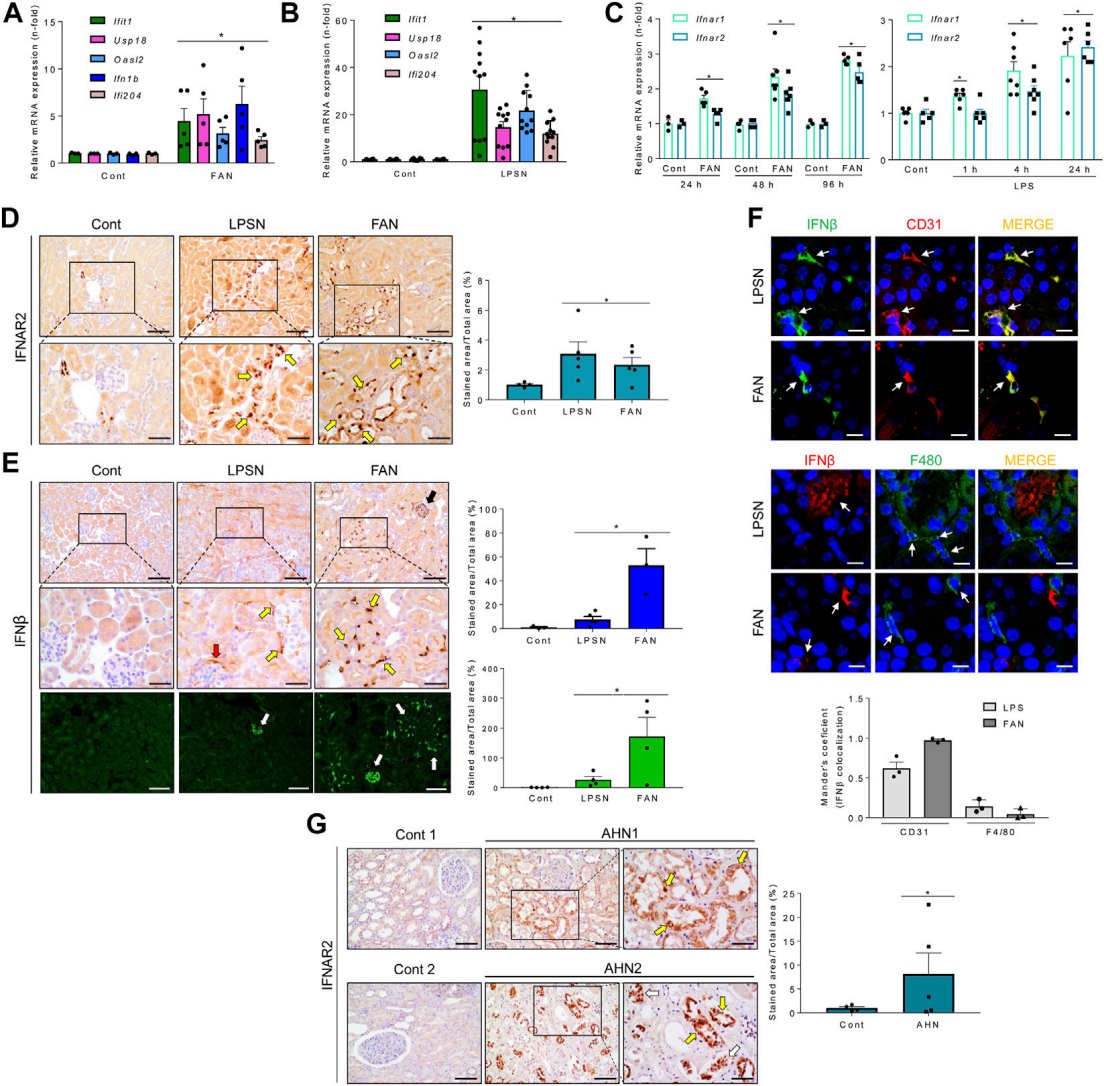
*In vivo* activation of the TI-IFN pathway in the kidney of mice or humans with kidney tubulointerstitial disease. **(A,B)** Kidney mRNA expression of ISGs (*Ifit1*, *Usp18*, *Oasl2*) and *Ifnb* measured by qRT-PCR in murine tubulointerstitial kidney injury induced by folic acid (FAN) **(A)** or LPS **(B)**. Bar charts represent the Mean ± SEM in control and kidney injured mice. *p ≤ 0.05 vs vehicle-injected control mice. **(C)** Mice with FAN (left) or LPSN (right) show higher renal IFNAR1 and IFNAR2 mRNA levels than controls (Cont), detected by qRT-PCR at different timepoints (LPSN: 1–24 h; FAN: 24–96 h). *p ≤ 0.05 vs control untreated animals (n = 3-7/temporal group). **(D)** Kidney IFNAR2 was overexpressed in LPS and FAN mice. Representative optical microscopy pictures show an increased tubular location of IFNAR2 detected by immunohistochemistry, especially in sections of intense tubular damage (boxed areas which are shown at higher magnification below). Yellow arrows point to damaged/dilated tubules expressing IFNAR2. The bar chart shows the signal quantification in the entire set of control and LPS-treated (LPSN, 24 h; *n* = 5) or folic acid-treated (FAN, 48 h; *n* = 4) animals. Results are expressed as the Mean ± SEM. *p ≤ 0.05 vs control animals without kidney injury. Original magnification ×200. Scale bar 100 µm. **(E)** Increased kidney IFNβ expression during LPSN and FAN. Representative optical microscopy pictures of immunohistochemical detection of IFNβ in LPS (*n* = 3) and FAN (*n* = 3) kidneys. Boxed sections and the corresponding magnified images below highlight areas of intense staining surrounding tubules (yellow arrows), the Bowman’s capsule (red arrow), and inside glomeruli (black arrow), suggestive of endothelial location. *p ≤ 0.05 vs control untreated group. Original magnification ×200. Increased IFNβ was also detected by confocal microscopy (white arrows). Scale bar 100 µm. **(F)** Confocal microscopy images of representative LPSN and FAN kidneys stained for IFNβ (green) and CD31 (red) (upper panel) and for IFNβ (red) and F4/80 (green) (middle panel). Endothelial cells expressing CD31, but not mononuclear phagocytes (F4/80), display a marked IFNβ signal. Colocalization was assessed by Mander’s overlap coefficient (MOC) (ImageJ Fiji, JACOP plugging). Bar chart represents the Mean ± SEM, *n* = 3 mice/group) (lower panel). White arrows indicate cells tracked through the IFNβ/CD31 or IFNβ/F4/80 fluorescence channels. Original magnification ×200. Scale bar 10 µm. **(G)** Increased IFNAR2 expression in acute human tubulointerstitial nephropathy (AHN). Representative optical microscopy pictures from renal biopsies (AHN1 and AHN2) show damaged tubules with strong tubular IFNAR2 staining. Boxed areas are enlarged below. *In situ* (yellow arrows) or detached (white arrows) tubular cells show high IFNAR content. The graph shows the Mean ± SEM of IFNAR2 signal in the entire set of patients. *p ≤ 0.05 vs control group, *n* = 5. Original magnification ×200. Scale bar 100 µm.

The increased gene expression of some of these ISGs, IFNβ, and IFNAR1/2, taken as reporters of the TI-IFN pathway activation, were validated by PCR in mice with both FAN and LPS-induced nephropathy (LPSN) (Figures 1A–C). Moreover, in FAN and LPSN mice, increased renal expression of IFNAR2 was detected by immunohistochemistry in kidney tubules (Figure 1D). By contrast, expression of IFNβ, which remained undetectable in control mice, was markedly increased in LPSN and FAN mice in the tubulointerstitial space (Figure 1E) where its fluorescence signal heavily overlaps with the fluorescence signal of endothelial cells (CD31) but hardly with the fluorescence signal of mononuclear phagocytic cells (F4/80) (Figure 1F). In addition, immunohistochemical IFNAR2 staining increased in tubular cells in patients with tubular injury, supporting the clinical relevance of this finding (Figure 1G).

### IFNβ activates TI-IFN and NF-κB signaling in cultured tubular cells

**FIGURE 2:**
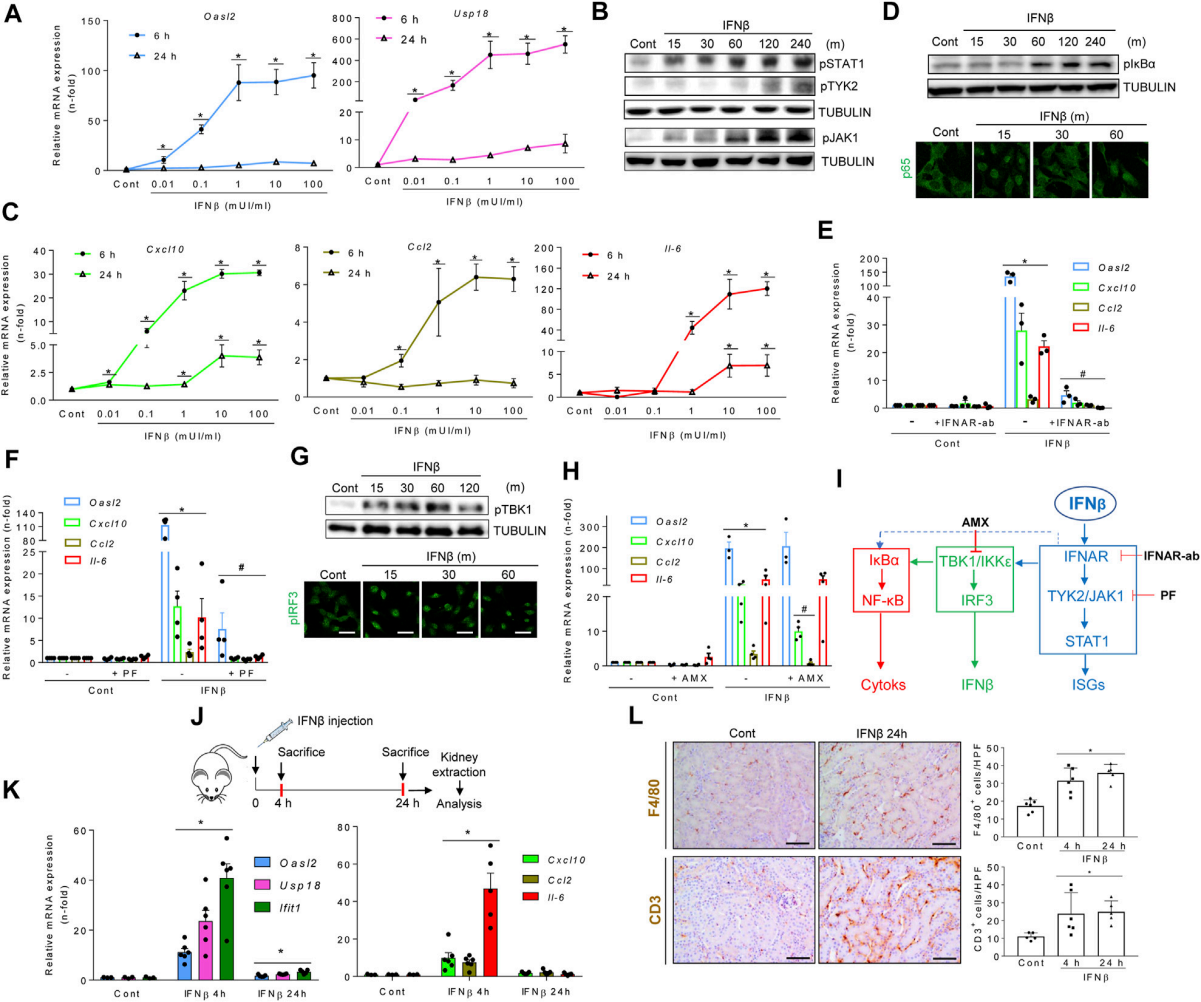
Type I interferons promote inflammation in renal tubular cells and kidney tissue. **(A)** Quantitative reverse transcription PCR (qRT-PCR) analysis of ISGs (*Oasl2, Usp18*) in MCT cells stimulated with increasing concentrations of IFNβ for 6 and 24 h. Values for mRNA were normalized to GAPDH expression. Data are expressed as the Mean ± SEM of three independent experiments. *p ≤ 0.05 vs untreated control cells. **(B)** Time-course (minutes, m) for the activation of the TI-IFN pathway. Representative western blot of phosphorylated isoforms of STAT1, JAK1 and TYK2 in MCT cells stimulated with 1.0 mUI/ml IFNβ. Tubulin was used as loading control. **(C,D)** Activation of the NF-κB pathway. **(C)** Cells were stimulated as indicated in A and the mRNA expression of cytokines (*Cxcl10, Ccl2, IL-6*) assessed by PCR. Data are Mean ± SEM of three independent experiments. *p ≤ 0.05 vs untreated control cells. **(D)** Representative western blot of phosphorylated IκBα (upper panel) and detection of nuclear p65 expression by immunofluorescence and confocal microscopy (green signal) (lower panel) in MCT cells stimulated with IFNβ for the indicated times (minutes, m). Original magnification ×400. Scale bar 10 µM. **(E,F)** Inhibition of the TI-IFN pathway by functional blockade of IFNAR **(E)** or pharmacological inhibition of TYK2/JAK1 **(F)**. MCT cells were preincubated with 10 μg/ml neutralizing anti-IFNAR antibody (IFNAR-Ab) or with 2.5 μM PF-06700,841 (PF) for 1 h before the addition of IFNβ for 6 h or 24 h *Oasl2* mRNA expression was assessed by RT-PCR after 24 h whereas *Cxcl10, Ccl2,* and *Il-6* gene expression were measured after 6 h. Results are shown as Mean ± SEM (*n* = 3). *p ≤ 0.05 vs untreated control cells; #*p* ≤ 0.05 vs IFNβ treatment. **(G,H)** Activation and functional analysis of TBK1/IKKε signaling. **(G)** MCT cells were stimulated with 1.0 mUI/ml IFNβ for the indicated times (minutes, m). Activation of TBK/IKKε was detected by their phosphorylated isoforms by western blot (pTBK1, upper panel) or confocal microscopy (pIRF3, lower panel). Original magnification ×400. Scale bar 10 µM. **(H)** Transcriptional response in MCT cells pretreated for 1 h with 50 µM amlexanox (AMX) before 1.0 mUI/ml IFNβ addition was assessed by qRT-PCR following 6 h (*Cxcl10, Ccl2, Il-6*) or 24 h (*Oasl2*). Data are Mean ± SEM of three independent experiments. *p ≤ 0.05 vs untreated control cells; ^#^ *p* ≤ 0.05 vs IFNβ-treated cells. **(I)** Molecular routes activated by IFNβ in tubular cells. IFNβ activates the canonical TI-IFN pathway leading to ISG expression by binding to IFNAR and recruiting TYK2/JAK1/STAT1 signaling (blue pathway). Downstream IFNAR or TYK2/JAK1, IFNβ also promotes the synthesis of proinflammatory cytokines and ISGs by activating the NF-κB (red pathway) and IRF3 (green pathway) transcription factors, respectively. Inhibiting TI-IFN signaling at IFNAR (IFNAR-Ab) or TYK2/JAK1 (PF-06700,841: PF) downmodulates the direct (blue pathway) or indirect (green pathway) pathways driving cytokine and IFNα/β gene expression, consistent with crosstalk between the TI-IFN and NF-κB pathways (discontinued blue line). Likewise, interfering with TBK1/IKKε activation (AMX) decreases both ISG and cytokine gene expression, disclosing a second crosstalk between the TBK1/IKKε signaling node and NF-κB. **(J-L)** Murine model of systemic IFNβ injection for evaluation of the renal response. **(J)** Experimental design for IFNβ administration to mice. **(K)** Time-course of mRNA expression of canonical ISGs (*Oasl2, Usp18, Ifit1*) and chemokines and cytokines (*Cxcl10, Ccl2, Il-6*) in kidneys from control or IFNβ-injected mice. Bar charts represent the Mean ± SEM for each gene (n = 5-6 mice/group). *p ≤ 0.05 vs control mice. **(L)** Immunohistochemical analysis and quantification of markers for mononuclear phagocytes (F4/80) (upper panel) and lymphocytes (CD3) (lower panel) in kidneys from control and IFNβ-injected mice. Representative microphotographs of immune cells in kidney tissue from control and IFNβ-injected mice at 24 h. The number of cells per high power field (hpf) was quantified and results were expressed as Mean ± SEM. *p ≤ 0.05 vs control untreated mice (n = 4-6 mice/group). Original magnification ×200. Scale bar 100 µm.

Whether TI-IFNs modulate inflammatory responses in tubular cells has not been firmly established. Thus, we next explored the impact of IFNβ on cultured murine proximal tubular MCT cells. IFNβ dose-dependently increased the mRNA expression of the canonical ISGs *Oasl2* and *Usp18*, peaking at 6 h and decreasing following 24 h, suggesting TI-IFN pathway activation (Figure 2A). Accordingly, IFNβ activated signaling downstream TI-IFN formation through JAK1, TYK2 and STAT1 phosphorylation (Figure 2B) and the transcription of NF-κB target cytokines like *Cxcl10* (also considered an ISG), *Ccl2,* and *Il-6*, with similar kinetics to canonical TI-IFN-responsive genes (Figure 2C). Supporting crosstalks between the TI-IFN and NF-κB pathways, IFNβ induced IκBα phosphorylation and the nuclear translocation of NF-κB/RELA (p65) (Figure 2D). IFNAR blockade with a specific anti-IFNAR1/2 neutralizing antibody (Figure 2E) or inhibiting TYK2/JAK1 with PF-06700841 (Figure 2F), dampened canonical ISG and cytokine transcription programs. IFNβ also induced phosphorylation of TBK1, IRF3 nuclear location (Figure 1G), and *Ifna/Ifnb1* mRNA expression (Supplementary Figure S1A), reflecting a positive activation loop in which IFNβ promotes the expression of TI-IFNs. Inhibition of both TBK1 and IKKε by amlexanox (Reilly et al., 2013) did not modify *Oasl2* mRNA levels, which is consistent with IFNAR signaling directly impacting the ISG response. However, amlexanox downregulated *Cxcl10* and *Ccl2* mRNA expression, without modifying *Il6* mRNA levels (Figure 2H), suggesting that IFNAR signaling engages TBK1/IKKε to promote the expression of NF-κB-dependent genes (Figure 2I). Indeed, IFNβ interacts with other key proinflammatory pathways because pre-exposure to IFNβ potentiated TWEAK-induced cytokine transcription to levels above an additive response (Supplementary Figure S1B). IFNα also elicited the ISG and cytokine responses in cultured tubular cells (Supplementary Figure S1C). Consistently, the IFNAR agonist RO8191 (Konishi et al., 2012) mimicked the strong IFNα/β-induced ISG response and the positive activation loop over the NF-κB (Supplementary Figure S1D). Thus, both TI-IFNs share a proinflammatory impact on tubular cells.

*In vivo*, a single dose of IFNβ (Figure 2J) transiently (peak 4 h) increased the kidney expression of ISGs (still mildly increased at 24 h) and proinflammatory cytokines (back to baseline at 24 h) (Figure 2K) resulting in tubulointerstitial inflammation characterized by infiltration by F4/80 + mononuclear phagocytes and CD3^+^ T lymphocytes that persisted for at least 24 h (Figure 2L). Collectively, these data establish that TI-IFNs promote proinflammatory and antiviral activities in cultured tubular cells and in the kidney *in vivo*.

### TWEAK and LPS activate the TBK1/IKKε/IRF3 and TI-IFN pathways

**FIGURE 3:**
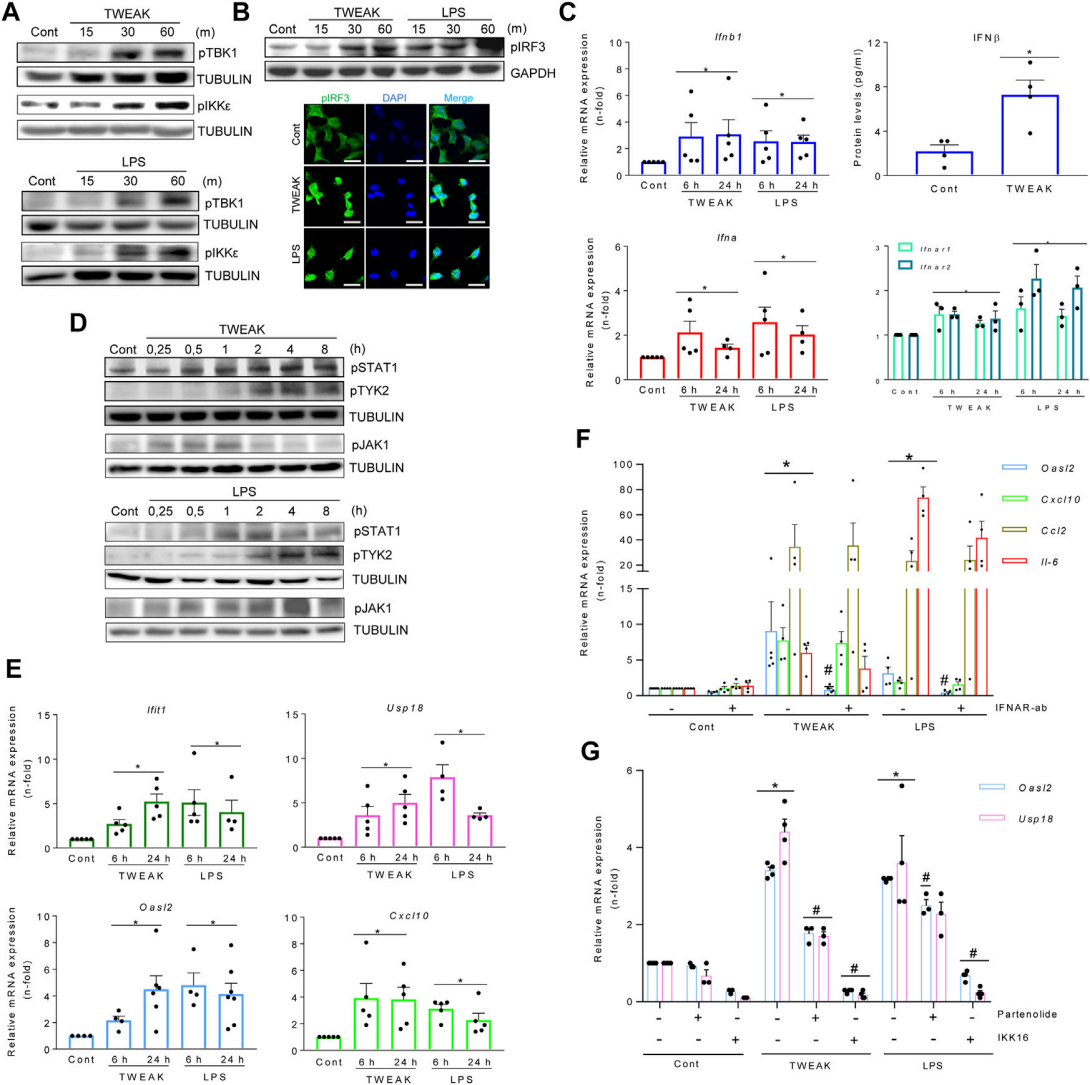
TWEAK and LPS activate the TBK1/IKKε and TI-IFN pathways in cultured kidney tubular cells and *in vivo* in the kidneys. **(A,B)** TWEAK and LPS activated the TBK1/IKKε/IRF3 pathway as assessed by phosphorylated TBK1 and IKKε detected by western blot **(A)** and phosphorylated IRF3 detected by western blot **(B, upper panel)** or immunofluorescence **(B, lower panel)** as compared with vehicle-treated control cells (Cont). Tubular MCT cells were stimulated with 100 ng/ml TWEAK or 1 μg/ml LPS for 15-60 min (m). Figures are representative of experiments repeated at least thrice. Original magnification ×400 in B (lower panel). Scale bar 10 µM. **(C–E)** TWEAK and LPS activated the TI-IFN pathway. **(C)** Quantitative RT-PCR for mRNA expression of *Ifna*, *Ifnb1*, and *Ifnar1/2* in MCT cells stimulated with TWEAK or LPS for 6 and 24 h, and TWEAK-dependent IFNβ secretion assessed in cell culture supernatants (24 h) by ELISA (right upper panel). Results are expressed as the Mean ± SEM of at least three individual experiments. *p ≤ 0.05 vs control untreated cells**. (D)** Representative western blots of increased levels of phosphorylated STAT1 (pSTAT1) and TYK2 (pTYK2) in total protein extracts from MCT cells stimulated with 100 ng/ml TWEAK or 1 μg/ml LPS. Stimulation times are expressed in hours **(H)**. **(E)** Extended gene expression study of samples analyzed in panel C shows that TWEAK and LPS also upregulated ISG genes (*Ifit1, Usp18, Oasl2, Cxcl10*). Results are the Mean ± SEM. *p ≤ 0.05 vs control untreated cells. **(F)** The contribution of the autocrine/paracrine recruitment of the TI-IFN pathway to TWEAK- and LPS-elicited responses in MCT cells was studied by blocking IFNAR with 10 μg/ml neutralizing anti-IFNAR antibody before stimulation with 100 ng/ml TWEAK or 1 μg/ml LPS. Gene expression was evaluated by q-RT-PCR after 6 h (*Cxcl10, Ccl2*) or 24 h (*Oasl2*). Results are expressed as Mean ± SEM of four or five experiments. *p ≤ 0.05 vs control untreated cells, ^#^ *p* ≤ 0.05 vs TWEAK- or LPS-stimulated cells. **(G)** Pharmacological inactivation of canonical IKKs limits TBK1/IKK-dependent ISG transcription in TWEAK- or LPS-stimulated MCT cells. Cultured MCT cells were pretreated for 1 h with chemical inhibitors of IKKα/β, namely 10 µM parthenolide (Parth) or 2.5 µM IKK16, before the addition of TWEAK or LPS for 24 h. Gene expression of ISGs (*Usp18, Oasl2*) was assessed by q-RT-PCR. Bar chart represents the Mean ± SEM. *p ≤ 0.05 vs control vehicle-stimulated cells and ^#^ *p* ≤ 0.05 vs TWEAK- or LPS-stimulated cells (*n* = 3/4).

We next studied whether proinflammatory stimuli known to cause kidney injury activate the TI-IFN pathway in MCT cells. TWEAK and LPS phosphorylate/activate TBK1, IKKε (Figure 3A), and IRF3 (which translocates into nuclei) (Figure 3B). These results are consistent with previously uncharacterized TWEAK-dependent noncanonical IKKs recruitment, and corroborated TI-IFN production following canonical LPS ligation to TLR4 (Uematsu & Akira, 2007).

Furthermore, increases in the IFNβ secretion in TWEAK treatments and in transcription levels of IFNα/β and IFNAR1/IFNAR2 in TWEAK and LPS treatments were found (Figure 3C). TWEAK and LPS also stimulated STAT1 and TYK2 phosphorylation (Figure 3D), i.e. signaling downstream of IFNAR, and correspondingly increased ISG mRNA expression at 6 and 24 h (Figure 3E). The autocrine/paracrine recruitment of the TI-IFN pathway was confirmed by IFNAR blockade (Figure 3F) or TYK2/JAK1 inhibition with PF-06700841 (Supplementary Figure S2A), as both prevented TWEAK- or LPS-induced ISGs transcription (*Oasl2*) without modifying the cytokine response.

Pretreatment with the IKKα/β inhibitor parthenolide limited long-lasting (120 m) TWEAK-induced TBK1 and IKKe phosphorylation (Supplementary Figure S2B) and both parthenolide and a second IKK inhibitor, IKK16, decreased the expression of TBK1/IKKε-dependent ISG genes in cultured tubular cells stimulated with TWEAK and LPS (Figure 3G). These results are consistent with reports of IKKα/β-mediated activation of TBK1/IKKε by NF-κB agonists such as TNFα, IL1β, or LPS, in BMDM and MEFs (Clark et al., 2011).

Overall, inflammatory stimuli that cause kidney injury, such as TWEAK and LPS, recruit the canonical NF-κB pathway (IKKα/β) to activate TBK1/IKKε and IRF3-mediated autocrine/paracrine loop of TI-IFN on IFNAR, leading to ISG transcription.

### TWEAK engages TLR4 to activate TBK1/IKKε and NF-κB

**FIGURE 4:**
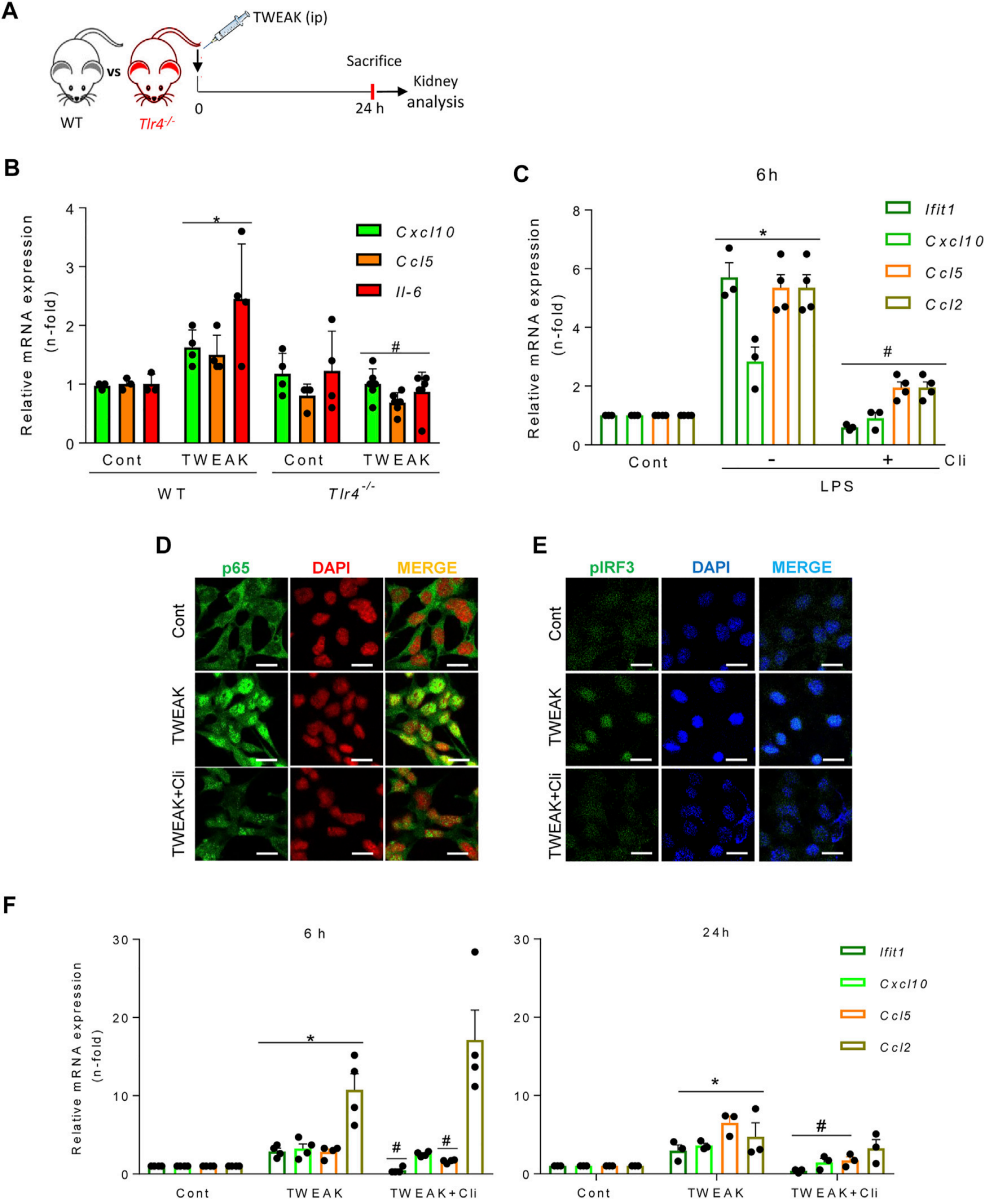
TWEAK transactivates TLR4 to recruit TBK1/IKKε signaling in tubular cells. **(A)** TWEAK was administered ip to wild-type (WT) or *TLR4* ^*−/−*^ mice and kidney gene expression was analyzed at 24 h. **(B)** The expression of proinflammatory chemokines (*Ccl5* and *Cxcl10*) shown to be sensitive to TBK1/IKKε/IRF3-dependent regulation in MCT cells was assessed by qRT-PCR. *p ≤ 0.05 vs WT control mice; ^#^ *p* ≤ 0.05 vs TWEAK-injected WT mice (*n* = 4 mice per group). **(C)** Pharmacological inhibition of TLR4 downregulates LPS-induced cytokine and ISG responses in cultured tubular cells. MCT cells were stimulated with 1.0 μg/ml LPS alone for 6 h or pretreated with 10 μM of the TLR4 blocker Cli-095 (Cli) for 3 h prior to LPS stimulation for 6 h *Ifit1*, *Cxcl10*, *Ccl5,* and *Ccl2* mRNA expression was evaluated by qRT-PCR. The bar graph shows the Mean ± SEM of four individual experiments. *p ≤ 0.05 vs control; ^#^ *p* ≤ 0.05 vs LPS treatment. **(D,E)** Inhibition of TWEAK-induced RELA/p65 and pIRF3 nuclear translocation by pharmacological blockade of TLR4 in tubular cells. MCT cells were stimulated with 100 ng/ml TWEAK for 20 min or pretreated with Cli-095 (Cli) for 3 h prior to TWEAK stimulation. TLR4 blockade prevented TWEAK-induced RELA/p65 (green fluorescence) nuclear translocation **(D)** as well as nuclear phosphorylated IRF3 content (pIRF3, green fluorescence) **(E)**. Nuclei were stained with DAPI. The figure shows typical images obtained in a representative experiment. Original magnification ×400. Bars 10 µm. **(F)** Downregulation of TWEAK-induced cytokine and ISG responses in cultured tubular cells by pharmacological blockade of TLR4. Cultured MCT cells were stimulated with 100 ng/ml TWEAK for 6 h (left panel) or 24 h (right panel) with or without pretreatment with Cli-095 (Cli). *Ifit1*, *Cxcl10*, *Ccl5,* and *Ccl2* mRNA expression was evaluated by qRT-PCR. Mean ± SEM (*n* = 3,4); *p ≤ 0.05 vs control; ^#^ *p* ≤ 0.05 vs TWEAK.

**FIGURE 5:**
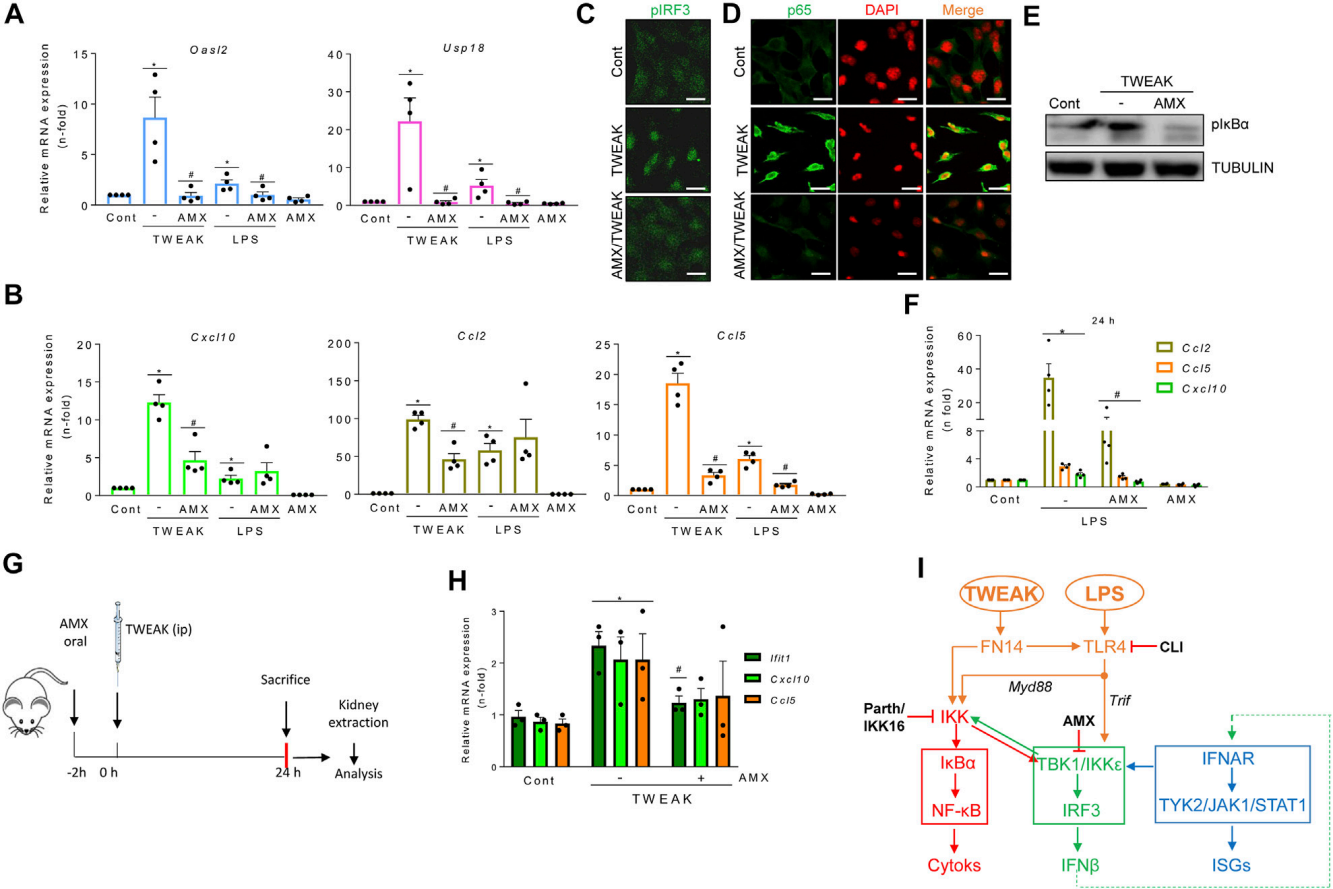
The TBK1/IKKε signaling node mediates TWEAK- and LPS-induced inflammation in cultured tubular cells and *in vivo* in the kidneys. **(A,B,F)** qRT-PCR assessed mRNA levels of ISGs and cytokines in MCT cells exposed to 100 g/ml TWEAK or 1 μg/ml LPS in the absence or presence of TBK1/IKKε inhibitor (50 μM amlexanox, AMX) added 1 h before stimuli. *Oasl2* and *Usp18* mRNA expression was evaluated at 24 h **(A)**. *Cxcl10*, *Ccl2* and *Ccl5* transcription was assessed at 6 h **(B)** or 24 h **(F)**. Values are Mean ± SEM. *p ≤ 0.05 vs control vehicle-stimulated cells and ^#^ *p* ≤ 0.05 vs TWEAK or LPS-stimulated cells (*n* = 4). **(C,D)** Representative microphotographs of nuclear phosphorylated IRF3 (pIRF3, green) **(C)** and p65 (green) **(D)** detected by immunofluorescence in MCT cells stimulated with TWEAK for 30 min in the absence or presence of AMX pretreatment for 1 h. Original magnification ×400. Scale bar 10 µM. **(E)** Representative western blot of pIκBα in cultured MCT cells stimulated with TWEAK in the same conditions as in D and **(E) (G,H)** Activation of TBK1/IKKε signaling *in vivo*. **(G)** TWEAK ip administration to mice with or without pretreatment with amlexanox (AMX) to evaluate the *in vivo* effects of TBK1/IKKε inhibition on TWEAK-induced kidney inflammation at 24 h. **(H)** The kidney expression of ISGs (*Ifit1*) and cytokines (*Cxcl10, Ccl5*) mRNA was assessed by qRT-PCR. *p ≤ 0.05 vs control vehicle-injected mice and ^#^ *p* ≤ 0.05 vs TWEAK-injected mice (n = 3 mice/group). **(I)** Tubular cell exposed to inflammatory stimuli (TWEAK, LPS) elicits antiviral responses that interact with proinflammatory pathways. TWEAK and LPS activate the canonical NF-κB pathway (red pathway) by activating the FN14 and Myd88-adapted TLR4 receptors, respectively (highlighted in orange). Inhibiting TBK1/IKKε with amlexanox (AMX) decreases the activation of TWEAK- and LPS-elicited NF-κB (red) and TI-IFN (blue) pathways downstream of TBK1/IKKε (green pathway). In addition, inhibition of canonical IKKs by parthenolide (parth) or IKK16 or blockade of direct LPS-mediated TLR4 activation (Trif adapted branch) or TWEAK-mediated transactivation of TLR4 by CLI095 also limited TBK1/IKKε activation.

Both TWEAK and LPS activated the TBK1/IKKε-dependent TI-IFN pathway in tubular cells. Therefore, we explored whether TWEAK recruited TLR4, the LPS receptor, to signal through TBK1/IKKε in TWEAK-treated WT and TLR4^−/−^ mice (Figure 4A). In WT mice, TWEAK administration upregulated the kidney mRNA expression of proinflammatory chemokines that are dependent on TBK1/IKKε signaling in cultured tubular cells (i.e., *Cxcl10* and *Ccl5*) (see below, Figure 5B) and this response was milder in *TLR4* ^*−/−*^ mice (Figure 4B). In cultured tubular cells, the TLR4 blocker CLI095 prevented the LPS-induced upregulation of cytokine and ISG gene expression, as expected for LPS binding and activation of TLR4 (Figure 4C). To further characterize the *in vivo* observations in *TLR4* ^*−/−*^ mice, we next tested the direct impact of CLI095 on TWEAK-stimulated cultured tubular cells. TLR4 blockade with CLI095 prevented the TWEAK-induced nuclear accumulation of RELA/P65 (Figure 4D) and phosphorylated IRF3 (Figure 4E) and the corresponding increase in *Ccl5* and *Ifit1* mRNA (6 and 24 h) as well as the persistence of *Cxcl10* mRNA upregulation (24 h), without modifying *Ccl2* mRNA levels (Figure 4F). Collectively, these data show that TWEAK can transactivate TLR4 to initiate antiviral and NF-κB-mediated transcriptional programs modulated by TBK1/IKKε/IRF3 signaling.

### TBK1/IKKε signaling mediates TWEAK- and LPS-induced renal antiviral and inflammatory responses

In exploring the participation of TBK1 and IKKε in antiviral and inflammatory responses elicited by proinflammatory stimuli other than IFNβ in tubular cells, amlexanox decreased TWEAK-induced ISG (Figure 5A) and cytokine (Figure 5B) mRNA expression. Consistent with these findings, amlexanox reduced nuclear p-IRF3 levels and NF-κB/p65 location (Figures 5C,D) and decreased p-IκBα levels (Figure 5E). Amlexanox also diminished the LPS-induced gene expression of ISGs and Ccl5 with a time course like TWEAK (Figures 5A,B), whereas *Cxcl10* and *Ccl2* downregulation was observed by 24 h (Figure 5F). In TWEAK-injected mice (Figure 5G), amlexanox also reduced kidney ISG and cytokine mRNA, consistent with cell culture observations (Figure 5H).

In summary, inflammatory mediators such as TWEAK and LPS recruit TBK1/IKKε in tubular cells through pathways that include canonical IKKs and TLR4. Thereby, TBK1/IKKε represents a signaling node located at the crossroad between inflammation and the antiviral response (Figure 5I).

**FIGURE 6:**
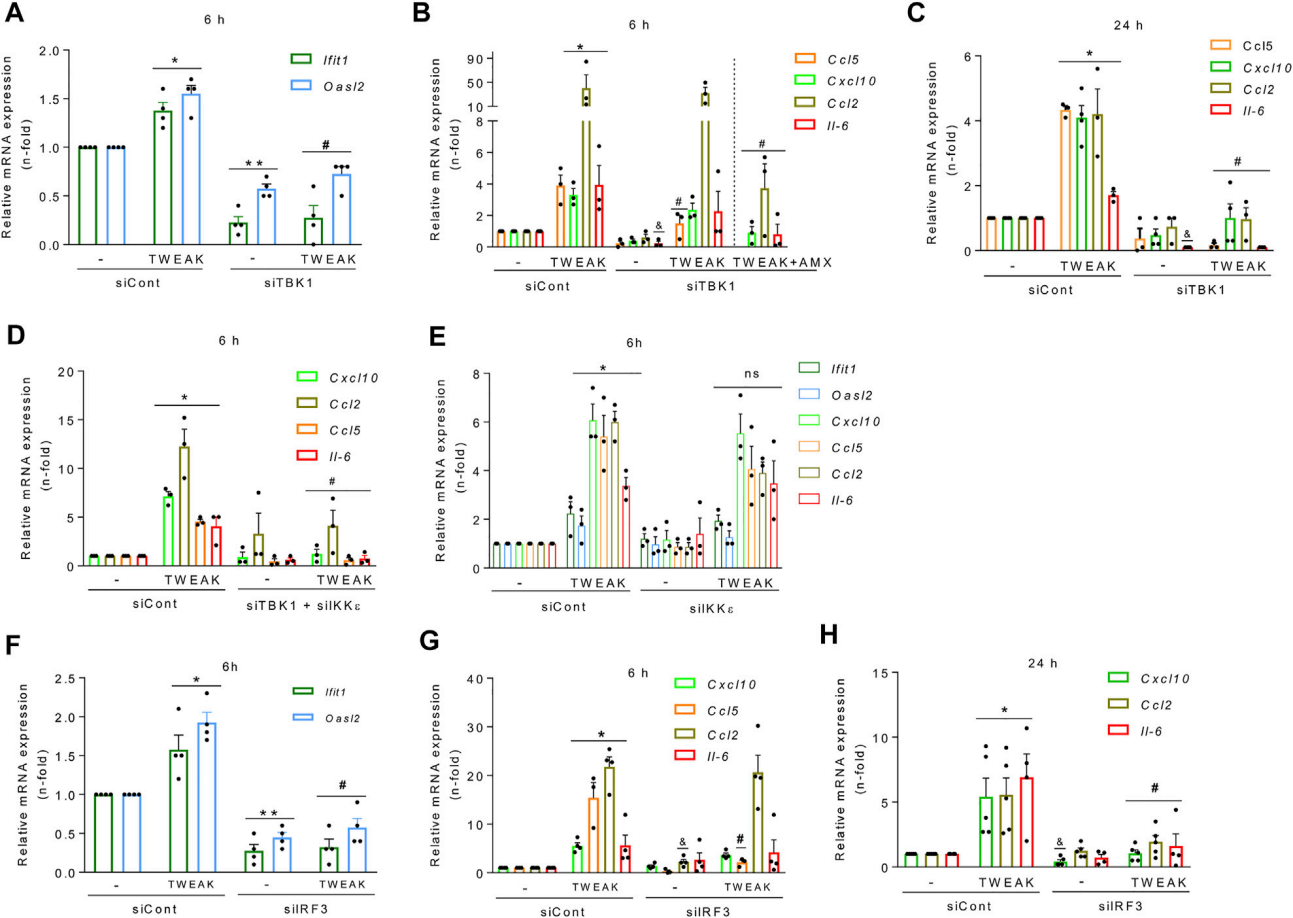
TBK1 and IRF3 inhibition prevent TWEAK-induced NF-κB and TI-IFN responses in tubular cells. MCT cells were transfected with specific TBK1 (siTBK1), IKKε (siIKKε), or IRF3 (siIRF3) siRNAs or with an irrelevant control siRNA control (siCont) before stimulation with vehicle or 100 ng/ml TWEAK for 6 h or 24 h to study gene expression. **(A–D)** TBK1 knockdown prevents antiviral and proinflammatory responses. **(A)** qRT-PCR of ISGs (*Ifit1, Oasl2*) after 6 h of vehicle or TWEAK stimulation following TBK1 silencing. **(B,C)** qRT-PCR of cytokines (*Ccl5, Cxcl10, Ccl2, Il-6*) in MCT cells stimulated with vehicle or TWEAK (alone or in the presence of amlexanox (AMX) for 6 **(B)** or with TWEAK for 24 h **(C)** following transfection with siCont or siTBK1. **(D)** qRT-PCR of ISGs and cytokines in MCT cells stimulated with vehicle or TWEAK for 6 h following transfection with siCont or with both siTBK1 plus siIKKε. **(E)** IKKε does not regulate NF-κB-dependent or ISG responses by itself. The same transcriptional profiles assessed in panels A and B were studied in MCT cells stimulated with vehicle or TWEAK for 6 h following transfection with siCont or siIKKε. **(F–H)** IRF3 knockdown downregulates TWEAK-induced antiviral and proinflammatory responses. Assessment of ISG (*Ifit1, Oasl2*) and cytokines (*Cxcl10, Ccl5, Ccl2, Il-6*) by qRT-PCR in MCT cells stimulated with vehicle or TWEAK for 6 h **(F,G)** or 24 h **(H)** following transfection with siCont or siIRF3. All results are expressed as the Mean ± SEM (n = 3/5); *p < 0.05, **p < 0.05 and ^and^ *p* < 0.05 vs siCont; ^#^ *p* < 0.05 vs siTBK1 or siIRF3.

Finally, we dissected the functional relevance of each component of the TBK1/IKKε/IRF3 pathway for TWEAK-induced NF-κB and antiviral response activation. Gene silencing reduced TBK1, IKKε, and IRF3 protein levels in tubular cells (≈90% at 48 h) (Supplementary Figure S3A). After further culture for 6 h, TBK1 silencing decreased the basal and TWEAK-induced mRNA expression of ISGs (Figure 6A), and *Ccl5* mRNA expression (Figure 6B) and secretion (Supplementary Figure S3B), but it did not modify Cxcl10, Ccl2, and Il-6 mRNA induced responses (Figure 6B). By contrast, together with *Ccl5*, they were suppressed at 24 h (Figure 6C), suggesting that recruitment of TBK1 promotes the persistence of TWEAK-induced inflammatory responses over time. Combining TBK1 silencing with amlexanox (Figure 6B) or the simultaneous silencing of TBK1 and IKKε thoroughly inhibited the TWEAK-induced mRNA expression of cytokines at 6 h (Figure 6D) suggesting that IKKε is required for early TWEAK-induced NF-κB activation after TBK1 engagement. However, IKKε silencing alone did not modify TWEAK-induced ISG or cytokine responses at 6 h (Figure 6E) or 24 h (Supplementary Figure S3C). Hence, coordinated TBK1 and IKKε activation is required for efficient activation of early TWEAK-induced inflammatory responses whereas only TBK1 is required to activate the ISG program.

Finally, like for TBK1 (Figure 6B), IRF3 silencing decreased basal and TWEAK-stimulated ISG mRNA expression (Figure 6F) but only the NF-κB-dependent *Ccl5* mRNA and protein upregulation at 6 h (Figure 6G, Supplementary Figure S3D), and the expression of *Cxcl10*, *Ccl2*, and *Il-6* mRNA at 24 h (Figure 6H), suggesting that the TBK1 axis contributes to the persistence of TWEAK-induced inflammatory responses over time.

### TBK1/IKKε signaling modulates tubular apoptosis induced by an inflammatory milieu including TWEAK

**FIGURE 7:**
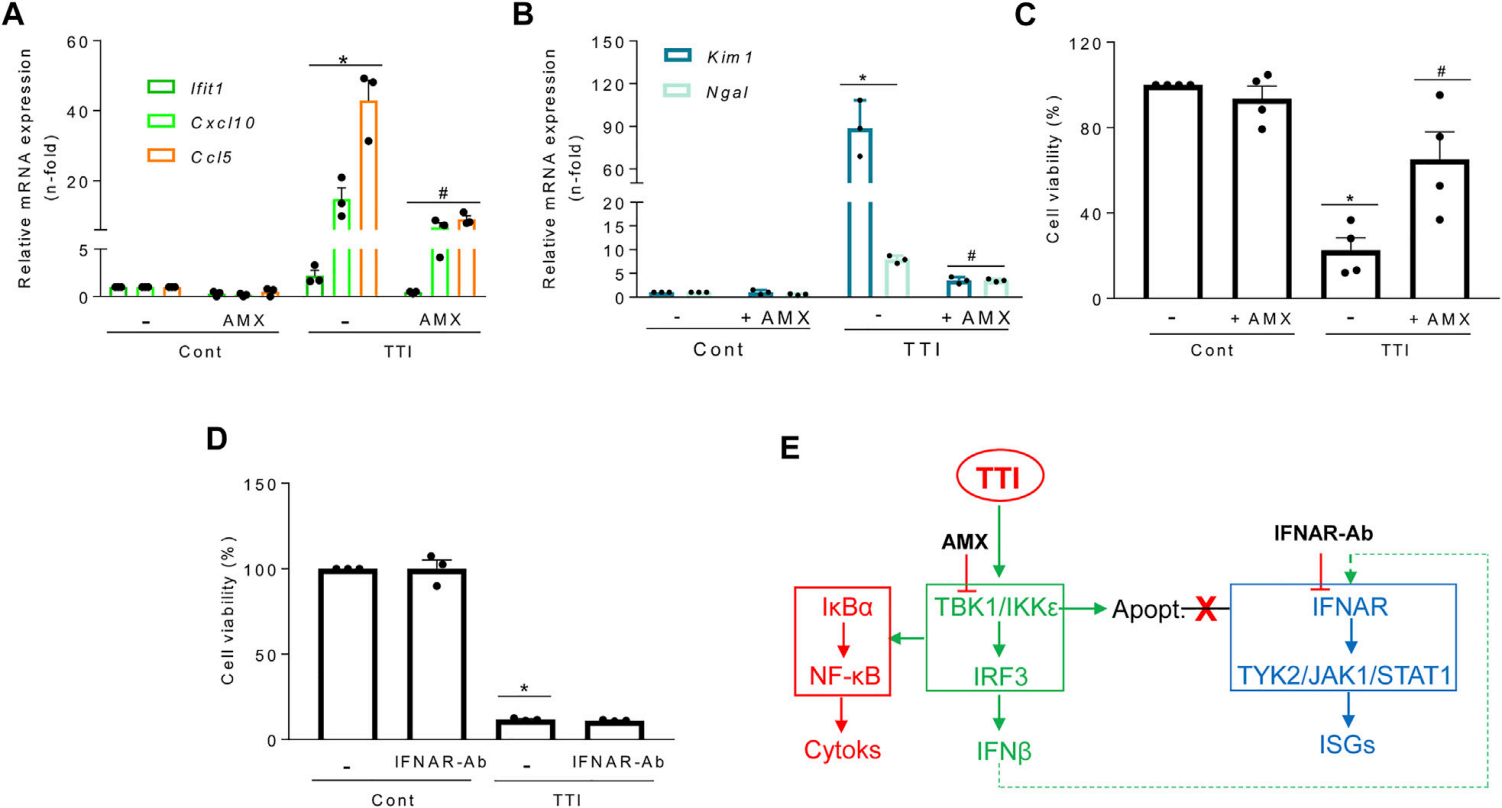
Role of the TBK1/IKKε tandem in gene expression and tubular cell death triggered by a complex proinflammatory microenvironment. Gene expression was assessed by qRT-PCR and cell viability by the MTT assay throughout all the experiments. Cultured MCT cells were pretreated for 1 h with 50 μM amlexanox (AMX) to inhibit TBK1 and IKKε simultaneously **(A–C)** or with anti-IFNAR neutralinzing antibody (IFNAR-Ab) **(D)** and then exposed to a mixture of cytokines composed of TWEAK/TNFα/IFNγ (TTI). **(A)** Amlexanox prevents the TTI-induced transcription of cytokines (*Cxcl10, Ccl5*) and ISG (*Ifit1*). Mean ± SEM (*n* = 3-4). *p ≤ 0.05 vs Control; ^#^ *p* ≤ 0.05 vs TTI. **(B)** Amlexanox reduced the mRNA expression of the tubular stress markers *Kim1* and *Ngal* in cells exposed for 6 h to TTI. Results are expressed as Mean ± SEM. *p ≤ 0.05 vs control; ^#^ *p* ≤ 0.05 vs TTI (*n* = 3). **(C)** Amlexanox reduced TTI-induced cell death (apoptosis) at 24 h. Mean ± SEM, *p ≤ 0.05 vs control; ^#^ *p* ≤ 0.05 vs TTI (*n* = 4). **(D)** Antibody-mediated IFNAR blockade (IFNAR-ab) does not prevent cell death (apoptosis) induced by TTI. Mean ± SEM (n = 3), *p ≤ 0.05 vs Control. **(E)** Functional consequences of TBK1/IKKε inhibition with amlexanox on inflammation, ISG transcription, and cell death in tubular cells. Amlexanox prevents the activation of the TBK1/IKKε signaling node (green) and subordinated cytokine (red) and ISG (blue) expression, as well as cell death (apoptosis) triggered by TTI which is not dependent on IFNAR blockade (red cross).

*In vivo*, cells are simultaneously exposed to multiple proinflammatory cytokines that may amplify tubular cell death. A cell microenvironment combining TWEAK, TNFα, and IFNγ (TTI) triggers both an inflammatory response and tubular apoptosis (Justo et al., 2006). Like TWEAK alone, TTI increased cytokine and ISG mRNA levels in cultured tubular cells and this was prevented by inhibition of TBK1/IKKε by amlexanox (Figure 7A). Amlexanox also decreased tubular stress and injury markers *Kim-1* and *Ngal* (Figure 7B) and cell death (Figure 7C) elicited by TTI, suggesting a role of TBK1/IKKε in driving inflammation-induced tubular cell death that is independent of IFNAR, as IFNAR blockade did not prevent TTI-induced cell death (Figure 7D). Altogether, these results support a role for TBK1/IKKε beyond mediating inflammation in also modulating cell death in tubular cells immersed in an inflammatory environment (Figure 7E).

### TBK1/IKKε and TI-IFN pathways modulate tubulointerstitial kidney inflammation and apoptosis *in vivo*

Based on the differential expression of genes related to the TI-IFN pathway in experimental and human kidney injury and the impact of TI-IFNs pathways on tubular cell biology, we addressed the impact of inhibiting TBK1/IKKε signaling or blocking IFNAR in sterile kidney tubulointerstitial inflammation and injury.

**FIGURE 8:**
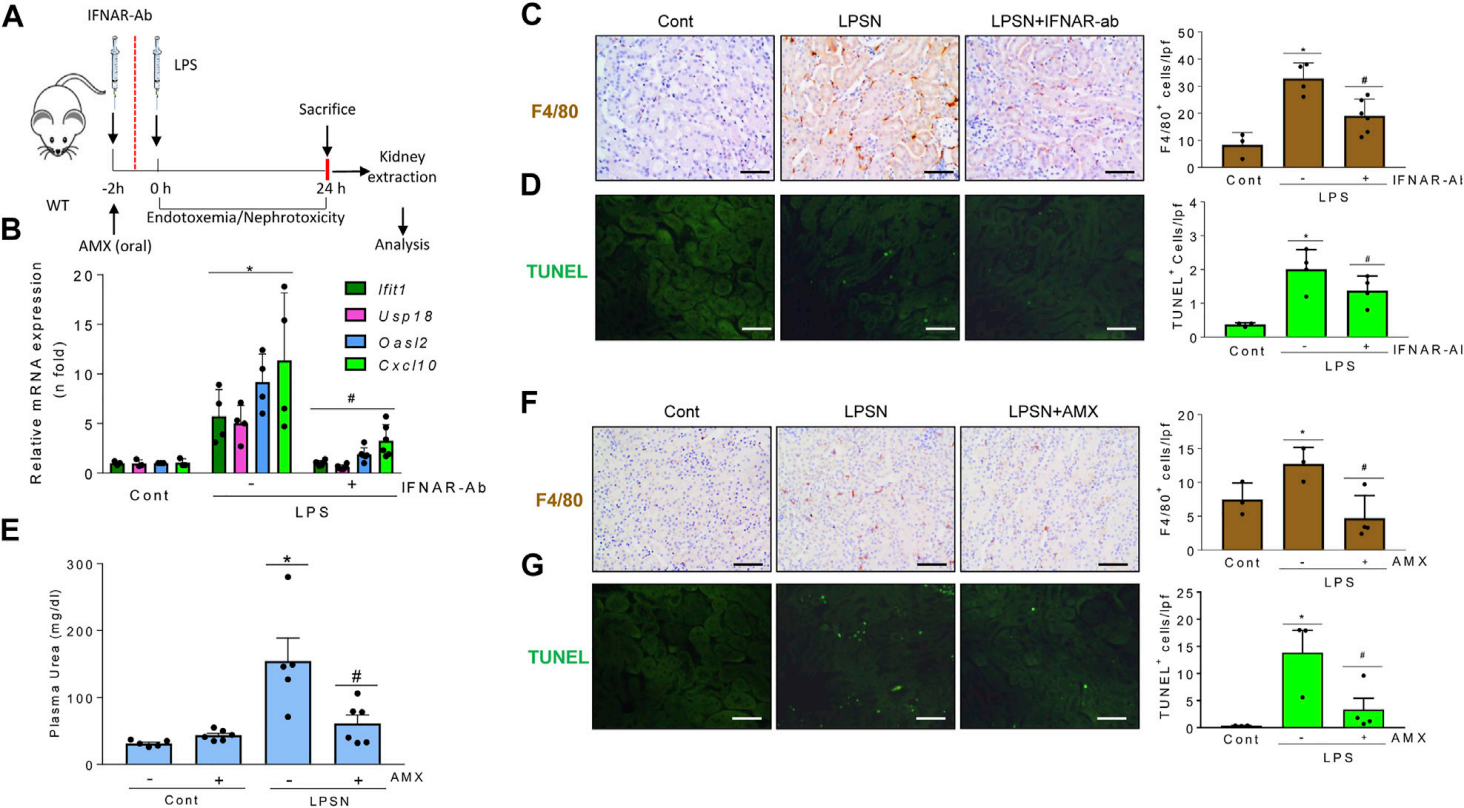
Signaling through TBK1/IKKε and IFNAR promotes kidney injury in LPS-induced nephropathy. **(A–G)** Functional relevance of TBK1/IKKε and IFNAR in the LPS nephropathy (LPSN) model. **(A)** Study design. Mice were pretreated with amlexanox (AMX) or anti-IFNAR neutralizing antibody (IFNAR-Ab) before LPS administration. **(B–D)** Impact of IFNAR blockade in LPSN. **(B)** qRT-PCR of kidney ISGs and cytokines (*Ifit1, Usp18, Oasl2, Cxcl10*). **(C)** Kidney infiltration by F4/80 + mononuclear phagocytes. **(D)** TUNEL+ dead cells. Mean ± SEM. *p ≤ 0.05 vs control; ^#^ *p* ≤ 0.05 vs LPS (*n* = 4). Original magnification ×200. Scale bar 100 µm. **(E–G)** Impact of TBK1/IKKε targeting by amlexanox (AMX) in LPSN. Amlexanox (AMX) decreased plasma urea levels **(E)** (Mean ± SEM. *p ≤ 0.05 vs control; ^#^ *p* ≤ 0.05 vs LPS; *n* = 5,6 mice per group); infiltration by F4/80 + mononuclear phagocytes **(F)**, and the number of TUNEL+ dead cells **(G)** in kidneys from mice with LPSN. Representative images and quantification. Mean ± SEM. *p ≤ 0.05 vs control; ^#^ *p* ≤ 0.05 vs LPS (*n* = 4 per group). Original magnification ×200. Scale bar 100 µm.

LPS administration to mice causes endotoxemia and nephrotoxicity. IFNAR blockade by anti-IFNAR antibodies (Figure 8A) decreased the LPS-induced upregulation of mRNA encoding several ISG, including *Cxcl10*, which targets Th1 lymphocytes and is involved in sepsis-associated kidney injury and other kidney disease conditions (Herzig et al., 2014; Gao et al., 2020) (Figure 8B). IFNAR blockade also decreased the interstitial infiltration by F4/80 + mononuclear phagocytes (Figure 8C), MPO + immune cells and Th1 (T-BET+) lymphocytes (Supplementary Figure S4), and restricted tubular cell death as assessed by TUNEL (Figure 8D). These results suggest that the TI-IFN pathway contributes to LPS-induced kidney inflammation and tubular cell death. We next inhibited TBK1 and IKKε with amlexanox (Figure 8A). Amlexanox protected from kidney dysfunction (Figure 8E), kidney infiltration by F4/80 + cells (Figure 8F), and cell death (Figure 8G).

**FIGURE 9:**
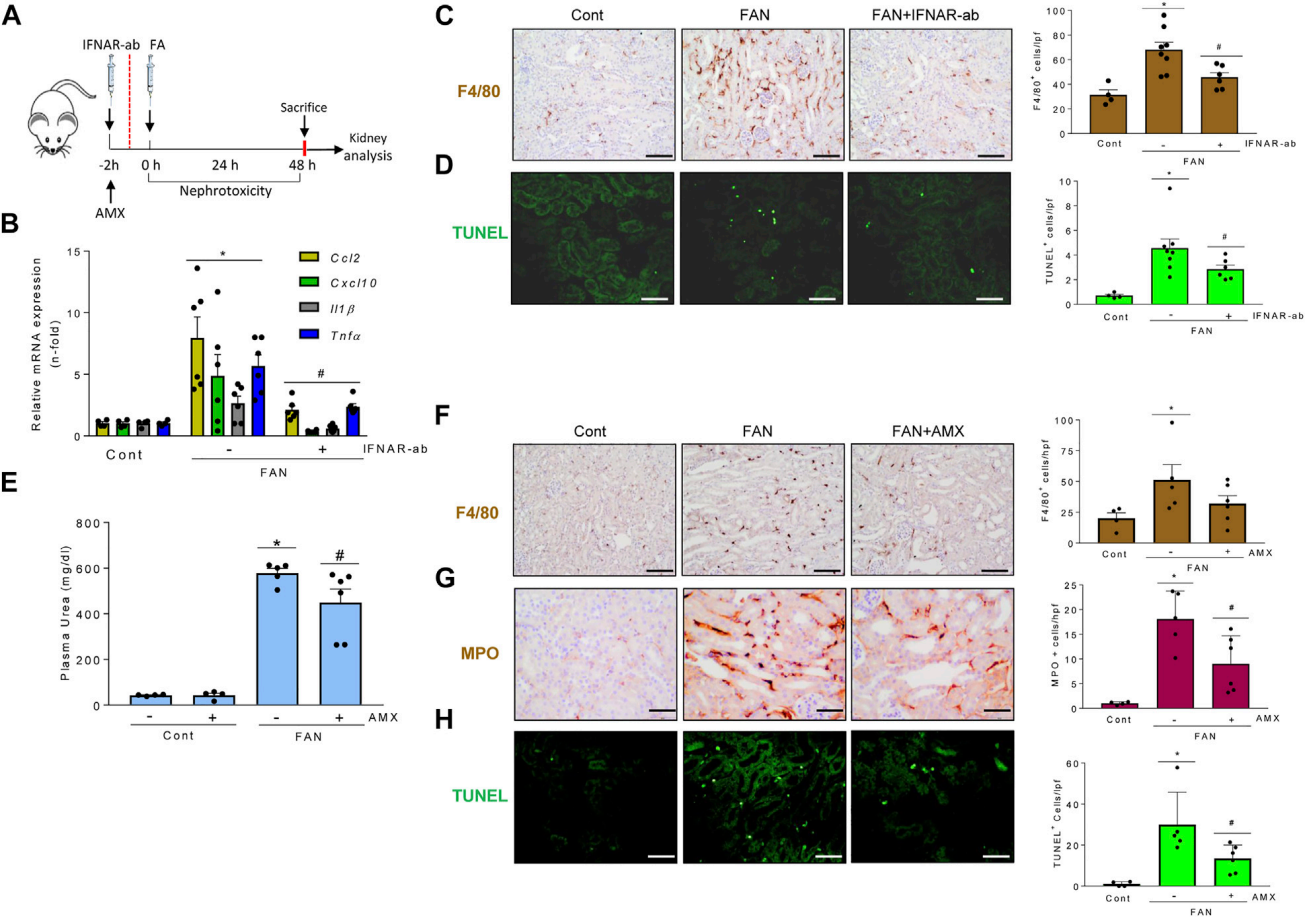
Signaling through TBK1/IKKε and IFNAR promotes kidney injury in folic acid-induced nephropathy. **(A–H)** Functional relevance of and IFNAR in the folic acid nephropathy (FAN) model. **(A)** Study design. Mice were pretreated with amlexanox (AMX), vehicle or anti-IFNAR neutralizing antibodies before folic acid administration. A second dose of anti-IFNAR was administered at 24 h. **(B–D)** Impact of IFNAR blockade in FAN. **(B)** qRT-PCR showed lower kidney expression of representative proinflammatory cytokines (*Ccl2*, *Cxcl10*, *Il1β*, *Tnf*) in mice with FAN and IFNAR blockade. **(C)** Kidney infiltration by F4/80 + mononuclear phagocytes. **(D)** TUNEL+ dead cells. Mean ± SEM. *p* < 0.05 vs Control, ^#^ *p* < 0.05 vs FAN (n = 4-8 mice per group). **(E–H)** Impact of TBK1/IKKε targeting by AMX in FAN. AMX decreased plasma urea levels **(E)**, infiltration by F4/80 + mononuclear phagocytes **(F)**, MPO + neutrophiles **(G)**, and the number of TUNEL+ dead cells **(H)** in kidneys from mice with FAN. Mean ± SEM. *p ≤ 0.05 vs control; ^#^ *p* ≤ 0.05 vs FAN; n = 4-6 mice per group.

In murine folic acid-induced nephropathy (FAN) (Figure 9A), IFNAR neutralization did not preserve renal function assessed by plasma urea levels (FAN: 245.9 ± 43.3 mg/ml; FAN/IFNAR-Ab: 274.0 ± 25.6 mg/dl; *p* = ns) but did decrease kidney cytokine and ISG mRNA expression (Figure 9B) and kidney infiltration by F4/80 + phagocytes (Figure 9C), supporting the contribution of the TI-IFN pathway to kidney inflammation. IFNAR blockade also decreased tubular cell death assessed by TUNEL (Figure 9D). Likewise, treatment with amlexanox (Figure 9A) protected from kidney dysfunction (Figure 9E), kidney infiltration by F4/80+ and MPO + cells (Figures 9F,G), and cell death (Figure 9H). Finally, activating the TBK1/IKKε/IRF3 and TI-IFN pathways from the top with DMXAA (Vadimezan), that mimics signaling of TBK1/IKKε initiated by viral dsDNA or bacterial cyclic dinucleotides (Roberts et al., 2007) increased renal cytokine mRNA expression and aggravated the course of FAN following 72-96 h by reducing mice survival. These results suggest deleterious effects of virus or bacteria-induced TBK1/IKKε/IRF3 and TI-IFN pathways over the course of renal injury (Supplementary Figure S4B).

Overall, results from nephrotoxic mouse modeling suggest that upstream (TBK1 and IKKε) and downstream (IFNAR) signals within the TI-IFN pathway promote kidney inflammation and injury.

## Discussion

We have now shown that TI-IFNs and proinflammatory factors involved in kidney injury, such as TWEAK and LPS, engage the TI-IFN pathway and associated noncanonical IKKs to activate NF-κB-dependent inflammation and cell death programs, therefore increasing the severity of inflammatory and nephrotoxic kidney injury. Specifically, amlexanox inhibition of TBK1/IKKε was identified as a novel therapeutic intervention for inflammatory and nephrotoxic tubulointerstitial kidney injury.

TI-IFNs are thought to contribute to the pathogenesis of viral or autoimmune glomerulonephritis. Glomerular cells express TI-IFN-target genes and cytokines in response to viral-like nucleic acids, and TI-IFNs induce apoptosis and inflammatory responses in these cells (Flür et al., 2009; Migliorini et al., 2013; Lorenz & Anders, 2015). TI-IFN signaling activates NF-κB to confer viral resistance, promote cell survival, and enhance inflammatory gene expression in nonrenal cells (Yang et al., 2000; Rubio et al., 2013; Piaszyk-Borychowska et al., 2019). In tubular cells, we demonstrated that IFNβ activates IFNAR and TYK2/JAK1 to recruit the NF-κB pathway, in a crosstalk that appears to be conserved for different species and cell lineages (Yang et al., 2000). Thus, the TBK1/IKKε signaling node sits at the crossroads of the TI-IFN and NF-κB signaling pathways and arises as a potential regulator of kidney inflammation and injury triggered by TI-IFNs. As maladaptive inflammation plays a key role in amplifying renal injury, NF-κB or related pathways that activate NF-κB, like the TBK1/IKKε/TI-IFN pathway which we now characterized, may contribute to interferon-related nephropathies, and become therapeutic targets (Sanz et al., 2010b; Markó et al., 2016; Gianassi et al., 2019; Song et al., 2019).

Tubular cells responded to TWEAK or LPS by activating the TBK1/IKKε/IRF3 pathway together with autocrine/paracrine TI-IFN signaling and ISG transcription. Mechanistically, TBK1 and IRF3 are required for TI-IFN pathway and ISG activation, whereas NF-κB activation depends on IKKε in immune cells (Balka et al., 2020). In contrast, TBK1 and IRF3 mediated NF-κB-dependent proinflammatory responses in tubular cells while IKKε did not contribute by itself to TWEAK-induced NF-κB and TI-IFN/ISG transcription programs. However, IKKε silencing or its pharmacological targeting with AMX in cells with inactive TBK1 allowed the suppression of the early TWEAK-induced NF-κB proinflammatory gene expression.

We also report that TI-IFN-related signaling modulates tubular apoptotic cell death. In cultured cell systems, the coordinated TBK1 and IKKε activities, or TBK1 by itself, inhibited RIPK1-dependent TNF-induced apoptosis or necroptosis (Lafont et al., 2018). Indeed, combined TBK1 and IKKε inactivation, or the loss of TBK1, sensitized to TNF lethality in lethal shock *in vivo* and the human TBK1 deficiency leads to autoinflammation driven by TNF-induced cell death (Lafont et al., 2018; Taft et al., 2021). Likewise, in tubular cells, the simultaneous inhibition of both TBK1 and IKKε by AMX regulated apoptosis, but, on the contrary, it protected tubular cells from TTI-induced apoptosis *in vitro* and *in vivo* kidney injury. Therefore, modulation of cell death by TBK1/IKKε inhibition, either by protecting or promoting it, is cell type- or pathology-specific.

Classical NF-κB activation by TWEAK depends on the engagement of the IKK complex and subsequent phosphorylation of IκBα (Poveda et al., 2013). Canonical IKKs mediated TNFα-induced TBK1 and IKKε phosphorylation and activation (Clark et al., 2011), in line with the present observation of TWEAK-induced TBK1/IKKε activation in tubular cells. Indeed, the TBK1/IKKε node was engaged by different families of inflammatory mediators (TWEAK, LPS, IFNβ) that share their convergence at NF-κB activation, resulting in reciprocal modulation between canonical and non-canonical IKKs. In addition, TWEAK also recruited the canonical NF-κB pathway through TLR4 transactivation, thus expanding the spectrum of receptors that are transactivated by TWEAK (Rayego-Mateos et al., 2013). TBK1/IKKε-mediated IKKβ phosphorylation had been already described in mouse MEFs, however, in these cells, IKKβ phosphorylation involved residues resulting in IKKβ inactivation rather than in IKKβ activation (Clark et al., 2011). In summary, as in other cell types, interactions between IKK family members were observed in tubular cells. However, the consequences of these interactions vary in a cell type-specific manner.

Evidence supporting a contribution of the TI-IFN pathway to kidney disease is incomplete and most related to immune-mediated glomerular injury, in which the contribution of the TI-IFN pathway to tubulointerstitial disease had been overlooked so far. In lupus patients, a pseudoviral immunity state associated with TI-IFN activation and high expression of an ISG signature is associated with more severe disease and nephropathy (Anders, 2009). In lupus-prone mice, interfering with IFNAR signaling improved nephritis. Moreover, clinical trials with the anti-IFNAR antagonistic monoclonal antibody Anifrolumab improved moderate to severe lupus (Felten et al., 2019). Anifrolumab is also undergoing clinical trials for lupus nephritis (Jayne et al., 2022). IFNAR^−/−^ mice are also protected from antibody-mediated glomerulonephritis or post-ischemic kidney injury (Freitas et al., 2011; Deng et al., 2021). We have now shown that in preclinical models characterized mainly by tubulointerstitial injury induced by LPS or folic acid, IFNAR blockade also decreased interstitial inflammation and tubular cell death, suggesting the existence of an intrarenal TI-IFN autocrine/paracrine loop involving renal resident or infiltrating immune cells. Consistent with this notion, in injured kidneys from mice with FAN and LPSN we identified increased IFNAR expression in the tubular epithelium together with a marked signal of IFNβ in CD31^+^ endothelial cells. IFNβ production by dermal endothelial cells and macrophages is thought to contribute to COVID-19 skin lesions, whereas IFNα from DCs promoted tubular injury in murine kidney ischemia-reperfusion (Deng et al., 2021; Domizio et al., 2022). Therefore, results in LPSN and FAN identify the endothelium as a key source of IFNβ that may bind to tubular IFNAR to amplify inflammation. Additionally, we have now demonstrated the contribution of TBK1/IKKε to both inflammation and cell death in injured kidneys, identifying drugs such as AMX as potentially kidney protective approaches.

TI-IFNs induced by viruses with renal tropism have been suggested to contribute to the development or increased severity of glomerulonephritis via direct TI-IFNs stimulation or secondary to release of proinflammatory or tissue-damaging mediators from glomeruli (Lai & Lai, 2006; Flür et al., 2009; Anders et al., 2010; Agrawal et al., 2021). SARS-CoV-2, a cause of acute kidney and glomerular injury, induces TI-IFNs and ISGs responses in tubular cells (Berthelot & Lioté, 2020; Omer et al., 2021). Nephrotoxicity, such as acute tubular necrosis and rejection, has also been observed following antiviral treatment with TI-IFNs in several conditions, as in kidney transplant recipients (Fabrizi et al., 2013). Recently, biopsy-proven thrombotic microangiopathy and focal glomerulosclerosis were reported in patients with IFNβ-associated nephropathy (Dauvergne et al., 2021). Although not reflected in the abstract, acute tubular necrosis was observed in 69% of the cases, being more frequent than glomerulosclerosis (Dauvergne et al., 2021). Data presented in the present manuscript suggest that tubular injury may be a primary event and not necessarily secondary to injury in other kidney structures. Furthermore, hyperactivation of the TI-IFN pathway and collapsing glomerulopathy has been described in STING-associated vasculopathy with onset in infancy (SAVI), an autoinflammatory disease resulting from gain-of-function *TMEM173/Sting* mutations (Abid et al., 2020). Again, interstitial inflammation and tubular injury were noted in the biopsy.

**FIGURE 10:**
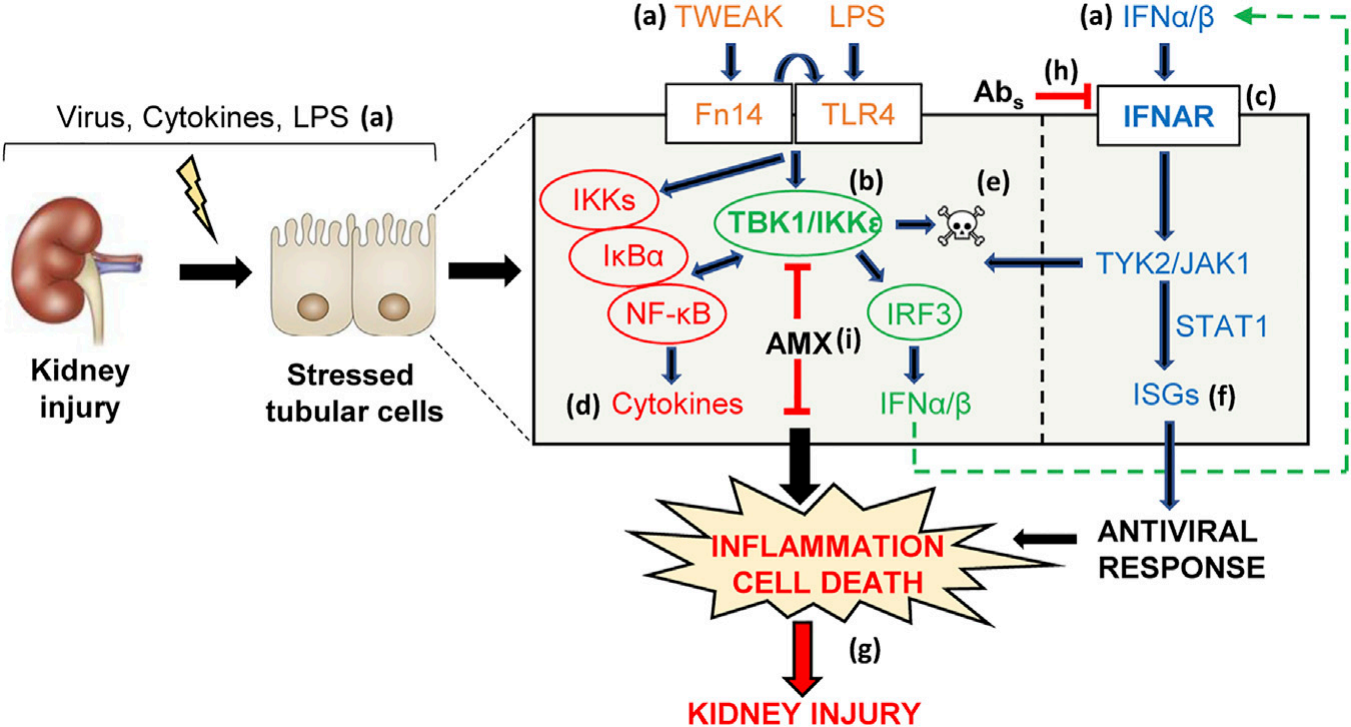
TI-IFN and related signaling pathways are potential pharmacological targets in kidney injury. Viral disease, pharmacological therapies with IFNα/β and endogenous (TWEAK, IFNα/β) or exogenous (LPS) proinflammatory inducers of kidney injury **(A)** may stress tubular cells that in turn activate adaptive innate immune mechanisms, including the intertwined TBK1/IKKε/IRF3 **(B)** and TI-IFN **(C)** pathways. Signaling through these pathways triggers NF-κB-dependent cytokine synthesis **(D)**, apoptosis **(E)**, and activates the ISG antiviral program **(F)**. Overall, these responses may amplify inflammation (cytokine storm), induce tubular cell loss (apoptosis), and contribute to kidney injury *in vivo* **(G)**. In this regard, immune-mediated inhibition of IFNAR with neutralizing antibodies **(H)** or the TBK1/IKKε signaling node with amlexanox (AMX) **(I)** was beneficial in decreasing renal inflammation and cell death **(G)**, thus allowing to identify TBK1/IKKε and IFNAR as novel therapeutic targets for nephroprotection.

In conclusion, TI-IFNs elicit intracellular signaling events in kidney tubular cells that interact with those elicited by other inflammatory mediators, thus contributing to tubulointerstitial kidney injury under different clinical scenarios, potentially including interferon therapy or the release of endogenous interferons during infection or sterile tissue injury (Figure 10). Specifically, the TBK1/IKKε node was identified as a druggable therapeutic target in kidney disease that acts as a hub linking diverse inflammatory stimuli with tubular cell death and inflammatory responses.

## Data Availability

The original contributions presented in the study are included in the article/Supplementary Material, further inquiries can be directed to the corresponding author.
