# Supplementary material for: Crosstalk between TBK1/IKKε and the type I interferon pathway contributes to tubulointerstitial inflammation and kidney tubular injury
Source: Front Pharmacol. 2022 Sep 23;13:987979. doi: 10.3389/fphar.2022.987979 (PMC9647636; doi:10.3389/fphar.2022.987979)
Supplement: Supplementary file 1 [file DataSheet1.PDF]

Supplementary Material

1 Supplementary Figures and Tables

1.1 Supplementary Figures

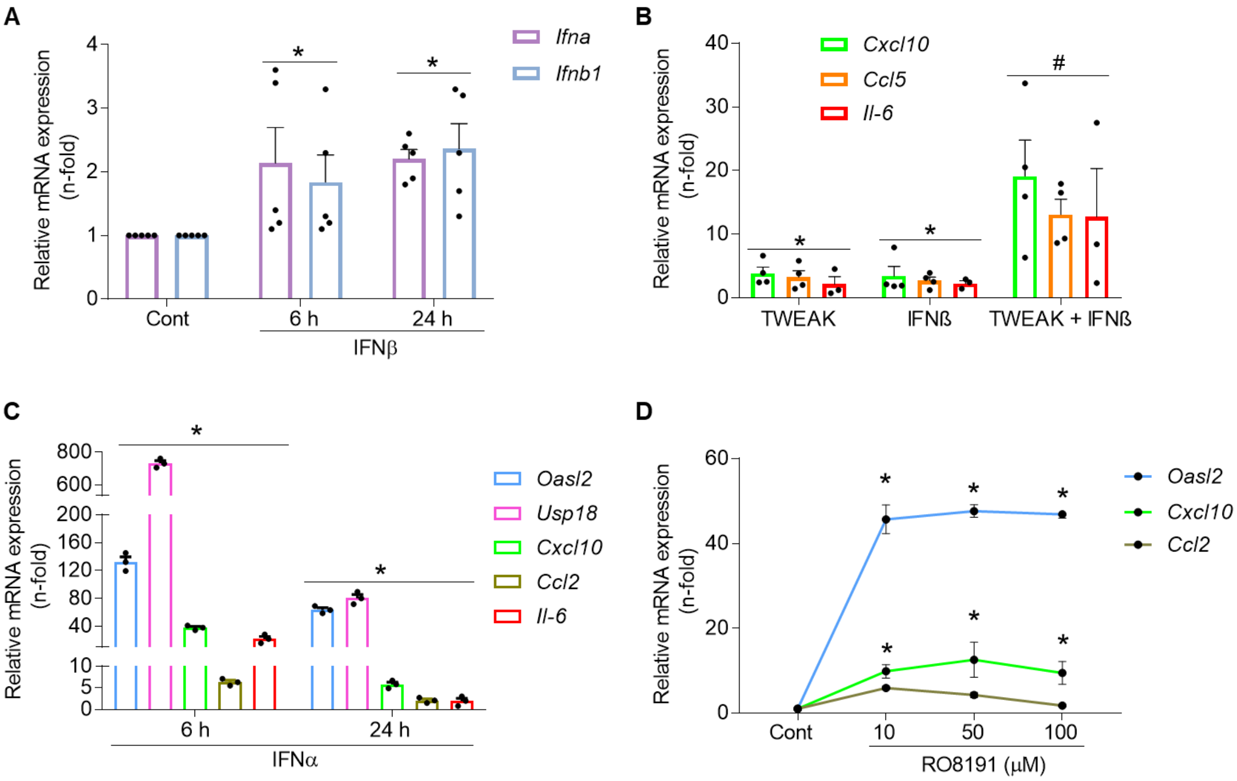

Supplementary Figure 1. Activity of TI-IFNs in tubular cells and kidney tissue. (A) Assessment of *Ifna* and *Ifnb* mRNA expression by qRT-PCR in MCT cells stimulated with 1000 UI/ml IFNβ for 6 h or 24 h. **(B)** Potentiation of proinflammatory responses by combining IFNβ and TWEAK. *Cxcl0*, *Ccl5*, and *Il-6* mRNA expression was evaluated by qRT-PCR in MCT cells stimulated with 1000 UI/ml IFNβ and then with 100 ng/ml TWEAK for 6 h. **(C)** Gene expression of ISGs (*Oasl2*, *Usp18*) and proinflammatory cytokines (*Il-6*), and chemokines (*Cxcl10*, *Ccl2*) assessed by qRT-PCR in MCT cells stimulated with 100 UI/ml IFNα for 6 h and 24 h. Results are expressed as Mean ± SEM. *$p \leq 0.05$ vs control; $^{\#}p \leq 0.05$ of combined stimulation (IFNβ + TWEAK) vs individual stimulation (IFNβ or TWEAK); n=3 to 5 individual experiments. **(D)** Induction of TI-IFN pathway and inflammatory genes by IFNAR agonism. MCT cells were stimulated for 6 h with the IFNAR agonist RO8191 and then the mRNA expression of *Oasl2*, *Cxcl10*, and *Ccl2* was quantified by qRT-PCR. *$p \leq 0.05$ vs control (n=3).

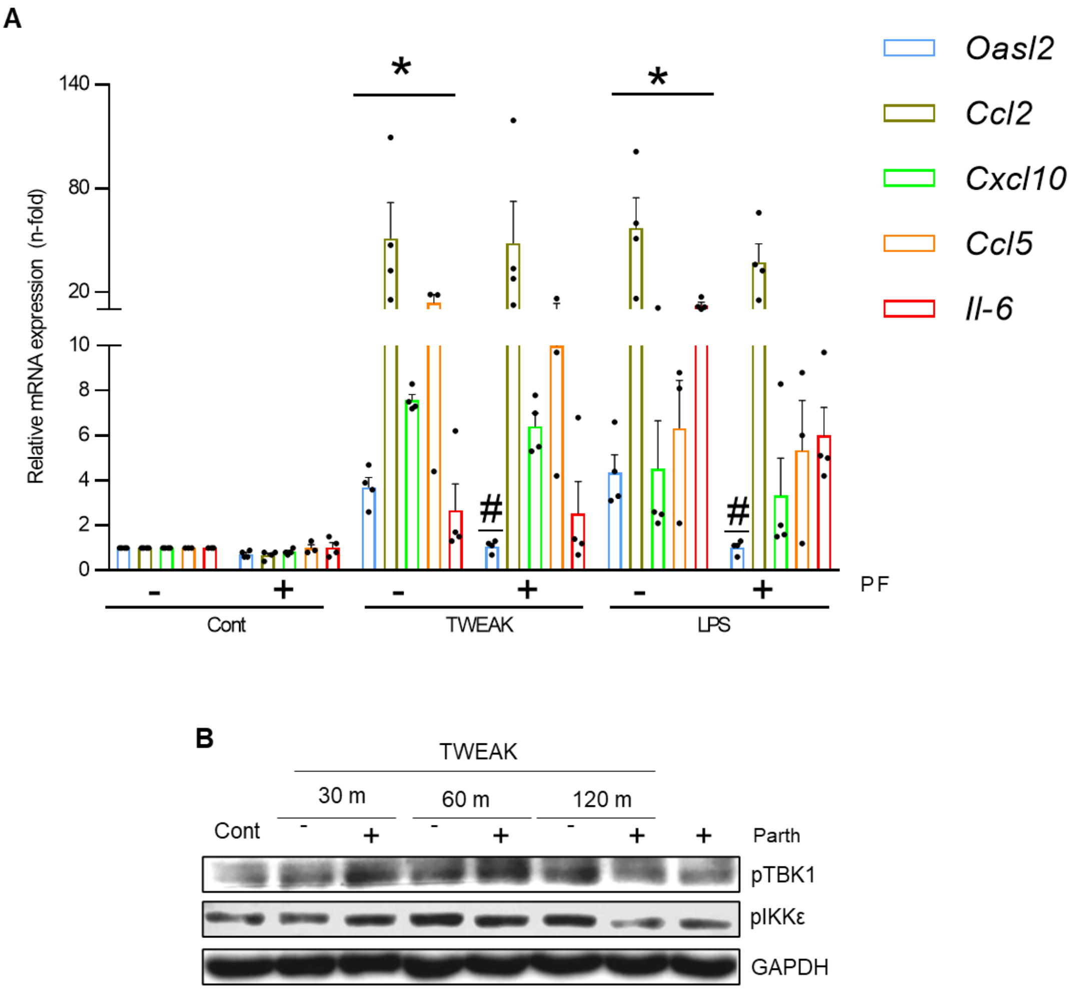

Supplementary Figure 2. Activation of upstream regulator signals of the overall TI-IFN pathway involved in proinflammatory stimulation of tubular cells. The activations of TYK2/STAT1 following IFNAR engagement and canonical IKKs upstream of the TBK1/IKKε tandem kinases were explored in MCT cells pretreated with inhibitors of TYK2/STAT1 (2.5 μM PF-06700841, PF) or IKK (10 μM parthenolide, Parth) for 30 m before the addition of 100 ng/ml TWEAK or 1 μg/ml LPS. **(A)** PF-06700841 inhibits the mRNA expression of the ISG *Oasl2*, without affecting the cytokine transcription profile. Results are expressed as Mean ± SEM (n=4). *p ≤ 0.05 vs control; $^{\#}$p ≤ 0.05 vs TWEAK or LPS. **(B)** Representative western blot showing that parthenolide limited TBK1 and IKKε phosphorylation over time.

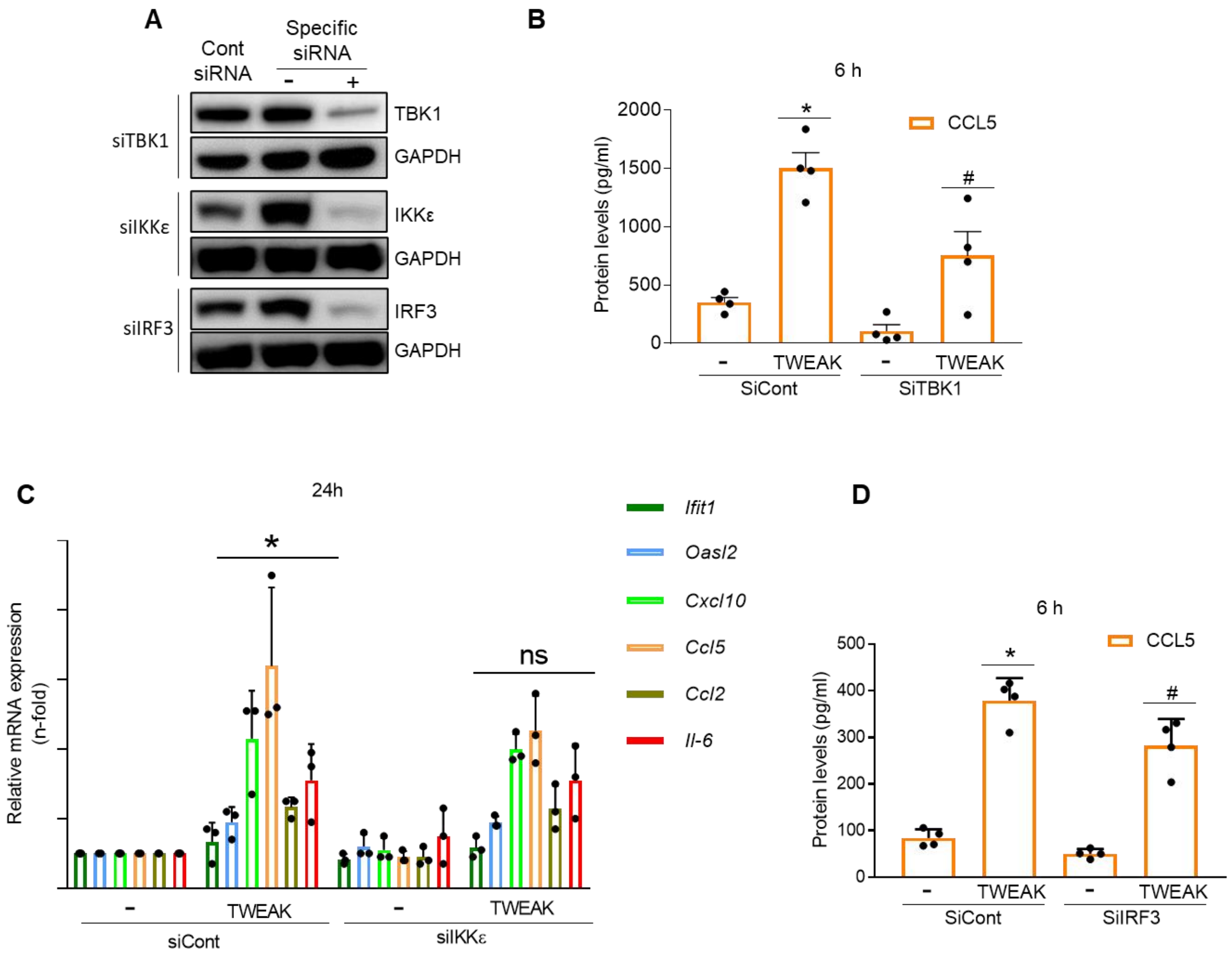

Supplementary Figure 3. Influence of genetic targeting of TBK1, IKKε, or IRF3 on TWEAK-induced ISG and cytokine expression in cultured tubular cells. (A) Efficiency of the silencing procedure for TBK1, IKKε, or IRF3 in tubular cells. Compared to control cells transfected with scrambled siRNA (siCont), specific siRNAs for TBK1 (siTBK1), IKKε (siIKKε), or IRF3 (siIRF3) decreased the expression of each of the proteins by approximately 90% after 48 h. **(B)** Silencing of TBK1 (siTBK1) in MCT cells prevented the synthesis and secretion of CCL5 assessed by ELISA in supernatants of cells stimulated with TWEAK for 6 h. Bar chart represents the Mean ± SEM (n=4). *p ≤0.05 vs siCont; $^{\#}$p ≤0.05 vs TWEAK treatment in cells transfected with siCont. **(C)** Silencing of IKKε did not regulate ISG and cytokine gene expression. siIKKε transfected cells stimulated with TWEAK for 6 h showed similar upregulation of ISGs and cytokine mRNA than cells transfected with a siCont and stimulated with TWEAK. *p ≤0.05 vs siCont. **(D)** Silencing of IRF3 (siIRF3) in MCT cells prevented the synthesis and secretion of CCL5 assessed by ELISA in supernatants of cells stimulated with TWEAK for 6 h. *p ≤ 0.05 vs siCont; $^{\#}$p ≤ 0.05 vs TWEAK stimulated cells transfected with siCont.

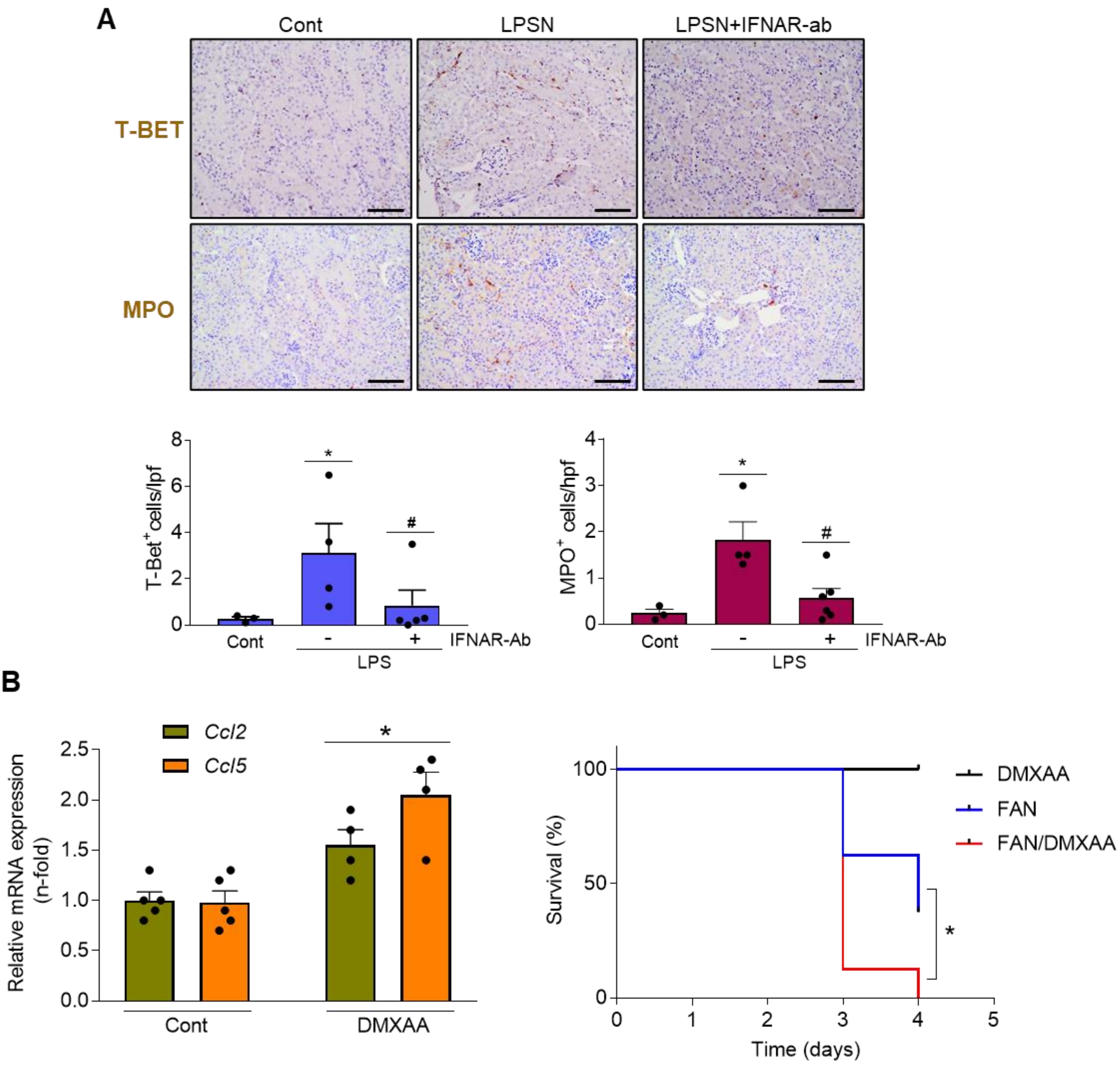

Figure S4. Involvement of IFNAR and TBK1/IKKε signaling in experimental tubulointerstitial nephropathy. A) Representative immunohistochemistry images and quantification show an increased kidney infiltration by T-BET+ Th1 lymphocytes and MPO+ myelomonocytic cells in mice with LPS nephropathy (LPSN) and its reduction by IFNAR blockade (LPSN+IFNAR-ab). Mean ± SEM, *$p \leq 0.05$ vs control; $^{\#}p \leq 0.05$ vs LPS (n=4-6/group). Representative images. Original magnification x 200. Scale bar 100 µm. **B)** Activation of the TBK1/IKKε pathway in mice with the viral/bacterial mimetic DMXAA aggravates the course of FAN. DMXAA activates *Ccl2* and *Ccl5* mRNA expression in mice treated with DMXAA for 96 h. Results are expressed as the Mean ± SEM; p ≤ 0,05 vs Cont untreated mice (left panel). Cotreatment with DMXAA reduces survival in mice with FAN (FAN/DMXAA) compared to animals with FAN that were left untreated (FAN). Mice that were treated with DMXAA alone, survived throughout the model length. * p ≤ 0,05 (FAN/DMXAA vs FAN) at 3 and 4 days after receiving the folic acid (rigth panel).

1.2 Supplementary Tables

Supplementary Table 1. Clinical characteristics of the patients.

Patient	***Sex***	***Age (years)***	***sCr (mg/dl)***	***Etiology***
1	F	55	8.7	Acute tubular necrosis
2	M	52	8.1	Ischemia
3	M	47	5.5	Transplant rejection
4	F	61	6.2	Renal vein thrombosis
5	M	71	6.9	Ureteral obstruction
Healthy	4 F	57.2 ± 9.2	7.1 ± 1.3	

Supplementary Table 2. Upstream regulators of the TI-IFN pathway with an absolute z-Score > 2.0 and p-value < 0.05. Values were calculated from differentially expressed genes in kidney transcriptomics of FAN mice versus control mice (24 h). Z-Score values were calculated by IPA and predict the activity of the identified regulators of the TI-IFN pathway.

UPSTREAM REGULATOR	ACTIVATION Z-SCORE	P-VALUE
Interferon alpha	6.468	8.09E-13
Ifnar	4.798	2.87E-12
Irf3	4.366	1.96E-10
Irf7	4.342	5.95E-08
Ifn type 1	3.238	0.96E-3
Ifnar1	2.817	3.19E-09
Irf5	2.526	1.38E-6
Ifn beta	2.471	2.59E-09

Supplementary Table 3. Upregulated interferon stimulated gene (ISG) signature expression in the kidney of mice with folic acid nephrotoxicity (FAN) at 24 h. Fold change versus control mice. Statistical significance was estimated through p-value (p) and false discovery rate (FDR), ns (not significant).

Gene symbol	Fold-change	p	FDR
IFI205	3.0	0.0002	0.0106
TMEM173	2.8	0.0001	0.0084
STAT3	2.3	0.0000	0.0055
CH25H	2.2	0.0003	0.0118
ISG20	2.2	0.0052	0.0454
ICAM1	2.0	0.0000	0.0049
GBP2	1.9	0.0002	0.0106
TAP1	1.7	0.0000	0.0049
SLFN9	1.7	0.0002	0.0097
ZBP1	1.7	0.0004	0.0121
IFIT1	1.7	0.0079	ns
PSMB8	1.6	0.0001	0.0068
SLFN1	1.6	0.0004	0.0125
SLFN4	1.6	0.0004	0.0126
ZC3HAV1	1.6	0.0016	0.0237
CXCL9	1.6	0.0023	0.0292
EGR1	1.6	0.0050	0.0445
OASL1	1.5	0.0001	0.0069
STAT1	1.5	0.0001	0.0087
OASL2	1.5	0.0003	0.0114
IRF7	1.5	0.0009	0.0178
IRF1	1.5	0.0006	0.0151
JAK1	1.5	0.0012	0.0204
IFI204	1.5	0.0040	0.0392
IFITM3	1.4	0.0005	0.0138
STAT2	1.4	0.0021	0.0276
MX2	1.4	0.0057	0.0479
EIF2AK2	1.4	0.0110	0.0726
MX1	1.4	0.0175	0.0959
USP18	1.4	0.0342	0.1486
ADAR	1.3	0.0041	0.0400
ISG15	1.3	0.0053	0.0459
DDX58	1.3	0.0055	0.0468
OAS1G	1.3	0.0087	0.0616
IRF9	1.3	0.0096	ns
IFI35	1.3	0.0145	0.0850
SLFN2	1.3	0.0297	0.1355
IFITM6	1.2	0.0064	ns
IFITM2	1.2	0.0079	ns

BST2	1.2	0.0114	ns
CGAS	1.2	0.0199	ns
IFIH1	1.2	0.0242	ns
